# Association between borderline dysnatremia and mortality insight into a new data mining approach

**DOI:** 10.1186/s12911-017-0549-7

**Published:** 2017-11-22

**Authors:** Yannick Girardeau, Anne-Sophie Jannot, Gilles Chatellier, Olivier Saint-Jean

**Affiliations:** 1 Biomedical Informatics and Public Health Department, Hôpital Européen G. Pompidou, Assistance Publique-Hôpitaux de Paris, Paris, France; 2 Sorbonne Universités, UPMC Univ Paris 06, UMR_S 1138, Centre de Recherche des Cordeliers, F-75006 Paris, France; 3 Université Paris Descartes, Paris, France; 4 Institut National de la Santé et de la Recherche Médicale (INSERM), Centre d’Investigations Cliniques, 1418 Paris, France; 5 Division of Geriatrics, Hôpital Européen G. Pompidou, Assistance Publique-Hôpitaux de Paris, Paris, France

**Keywords:** Hyponatremia, Hypernatremia, Borderline dysnatremia, Sodium, In-hospital mortality, Electronic health record, Phenome-wide association analysis

## Abstract

**Background:**

Even small variations of serum sodium concentration may be associated with mortality. Our objective was to confirm the impact of borderline dysnatremia for patients admitted to hospital on in-hospital mortality using real life care data from our electronic health record (EHR) and a phenome-wide association analysis (PheWAS).

**Methods:**

Retrospective observational study based on patient data admitted to Hôpital Européen George Pompidou, between 01/01/2008 and 31/06/2014; including 45,834 patients with serum sodium determinations on admission. We analyzed the association between dysnatremia and in-hospital mortality, using a multivariate logistic regression model to adjust for classical potential confounders. We performed a PheWAS to identify new potential confounders.

**Results:**

Hyponatremia and hypernatremia were recorded for 12.0% and 1.0% of hospital stays, respectively. Adjusted odds ratios (ORa) for severe, moderate and borderline hyponatremia were 3.44 (95% CI, 2.41–4.86), 2.48 (95% CI, 1.96–3.13) and 1.98 (95% CI, 1.73–2.28), respectively. ORa for severe, moderate and borderline hypernatremia were 4.07 (95% CI, 2.92–5.62), 4.42 (95% CI, 2.04–9.20) and 3.72 (95% CI, 1.53–8.45), respectively. Borderline hyponatremia (ORa = 1.57 95% CI, 1.35–1.81) and borderline hypernatremia (ORa = 3.47 95% CI, 2.43–4.90) were still associated with in-hospital mortality after adjustment for classical and new confounding factors identified through the PheWAS analysis.

**Conclusion:**

Borderline dysnatremia on admission are independently associated with a higher risk of in-hospital mortality. By using medical data automatically collected in EHR and a new data mining approach, we identified new potential confounding factors that were highly associated with both mortality and dysnatremia.

**Electronic supplementary material:**

The online version of this article (10.1186/s12911-017-0549-7) contains supplementary material, which is available to authorized users.

## Background

Dysnatremia is one of the most frequent electrolyte disorders in adult patients admitted to hospital [1]. Plasma sodium concentration is essentially determined by plasma water intake and loss (in urine, feces, and sweat) and the water content of the plasma is finely regulated by a system including sensory organs (e.g. the carotid receptor and hypothalamus), vasopressin and the kidney. Sodium is the main factor determining plasma osmolality. Even small variations of plasma sodium concentration lead to the movement of water between intracellular and extracellular spaces, with a potential clinical impact.

The consensus definition of hyponatremia is a serum sodium concentration of less than 135 mmol/L. As water moves from body compartments with a lower osmolality to those with a higher osmolality, hyponatremia induces the movement of water into cells, leading to edemas of the brain and other tissues. In addition to neurological complications [2], several epidemiological studies have identified an association between hyponatremia and mortality in hospitalized patients [3–5], the general population [6, 7] and patients with specific diseases, including heart failure [8, 9], liver cirrhosis [10, 11], pulmonary embolism [12], pulmonary hypertension [13, 14], pneumonia [15, 16], chronic kidney disease (CKD) [17, 18] and myocardial infarction [19, 20].

The consensus definition of hypernatremia is a serum sodium concentration exceeding 145 mmol/L, and this condition is far less frequent than hyponatremia [21]. Hypernatremia is caused principally by water loss leading to plasma hyperosmolality, resulting in the dehydration of cells [22]. People with altered thirst sensation (including patients with specific hypothalamic lesions, the elderly and patients with altered mental status) or with difficult access to water are particularly exposed to the risk of severe hypernatremia. The symptoms are often not specific, particularly for intermediate levels of hypernatremia (e.g. thirst, muscle weakness, anorexia, nausea, vomiting and altered consciousness) but severe hypernatremia can lead to brain shrinkage potentially associated with cerebral bleeding, subarachnoid hemorrhage and death [22].

Several recent studies have identified a potential association between mortality and dysnatremia, even for small variations of sodium concentration [3–5, 18, 23, 24]. However, most of these studies were conducted in specific cohorts and it remains unclear whether the patients concerned died due to the effects of dysnatremia or from the underlying diseases causing the dysnatremia [25, 26].

Real-life care data from Electronic Health Record (EHR) can be used to identify or confirm unknown disease correlation [27, 28]. This automatically captured health data offers new research approach. Methods and tools, known as data mining, have been developed over the years to analyse a large collection of data [29]. In 2010, a new protocol, phenome-wide association analysis (PheWAS) was developed to identify potential correlation between many phenotypes, called the phenome, and a given genetic variant [30]. After the first proof of concept, PheWAS was also used to identify new potential correlation between non-genetic target and phenotypes [31, 32]. To our knowledge, none has performed such method to explore methodically all the potential counfonders from EHR.

Our objective was (i) to assess the prevalence of dysnatremia in all patients admitted to hospital, (ii) to identify new confounders through a PheWAS analysis and compare them to classical confounders identified in the literature, (iii) to confirm the impact of dysnatremia on in-hospital mortality after adjustment for classical and new potential confounding conditions.

## Methods

### Data source

The Hôpital Européen Georges Pompidou (HEGP) is a 795-bed quaternary care hospital. Data were extracted from the HEGP clinical data warehouse (CDW), which prospectively collect all data from the electronic health record (EHR) established at the creation of the hospital in 2000.

### Study design and definitions

We conducted a retrospective observational study, extracting data for patients admitted to the HEGP between January 1, 2008 and June 31, 2014. We didn’t take data prior to 2008 as the coding of ICD-10 codes was not optimal before 2008 in our institution. We included patients having a stay fulfilling the following criteria: at least one serum sodium concentration determined the day of admission and a stay lasting at least 2 days. We used the same categories of sodium concentration as Funk et al. [5]: normal serum sodium concentration (135 ≤ [Na] ≤ 145 mmol/L) was used as the reference category. Hypernatremia was defined as borderline (145 < [Na] ≤ 150 mmol/L), mild (150 < [Na] ≤ 155 mmol/L) or severe ([Na] > 150 mmol/L) and hyponatremia was defined as borderline (130 ≤ [Na] < 135 mmol/L), mild (125 ≤ [Na] < 130 mmol/L) or severe ([Na] < 125 mmol/L). Serum sodium determinations were not corrected for the effects of hyperglycemia.

### Data extraction

As patients may have been hospitalized more than once, with normal, high or low sodium levels, we extracted patients as follows, to ensure that each patient corresponded to a unique observation in the dataset: 1) we first extracted patients encountered with severe hypernatremia; 2) we then extracted patients encountered with mild hypernatremia, excluding the patients already selected in the first step; 3) we extracted patients encountered with borderline hypernatremia, excluding the patients selected in the first two steps. We then applied the same process for hyponatremia. Finally, we extracted the first encounters with patients whose sodium levels were normal. We provide the SQL code extraction in Additional file 1.

### Demographic characteristics and Comorbidities

We extracted the following data from the HEGP-CDW for each included encounter: age, sex, duration of hospital stay, hospital admission category (via the emergency department vs. others), any surgery during the stay, intensive care unit (ICU) stay, palliative care, dialysis procedures and ICD-10 codes. We determined the ICD-10 adaptation of the Charlson comorbidity index [33]. We recorded vital status at hospital discharge.

### Statistical analysis

Binary variables are reported as numbers and frequencies (%); continuous variables are expressed as means and standard deviations and variables with skewed distributions are expressed as medians and interquartile ranges (IQRs). We used chi-squared tests to compare frequencies between sodium concentration categories and Fisher’s exact test, as appropriate. We compared medians with Wilcoxon signed-rank tests. We first assessed the association between mortality and each serum sodium concentration category at admission. We also assessed the associations between classical potential confounding factors (i.e.: age, sex, duration of hospital stay, number of diagnosis codes, hospital admission via the emergency department, ICU stay during hospitalization, palliative care, dialysis, dementia and the Charlson comorbidity index) and serum sodium concentration category with ANOVA, and the associations of these factors with mortality, using a logistic regression model. We categorized age, Charlson index and serum sodium level. We retrieved all potential confounding factors associated with both mortality and serum sodium concentration category. We adjusted for these factors in a multivariate logistic model in which these variables and serum sodium concentration category were treated as explanatory variables and in-hospital mortality was treated as the dependent factor.

The formula used for the multivariate models was.

Logit (Death) = β0 + β_1_ (Dysnatremia/Normal Serum Sodium level)+ Σ_i_ β_i_ x Confounding factors.

where confounding factors are those extracted in the literature (“classical model”), those extracted from PheWAS (“PHeWAS model”) or both (“Final model”).

### Phenome-wide association analysis

We performed a phenome-wide association study (PheWAS) to identify new potential confounding factors driving the association between borderline dysnatremia and mortality. PheWAS is based on the testing of associations between traits and all ICD-10 billing codes, with the aim of identifying associations that have never been described [31]. We performed three PheWAS analyses to identify new potential confounding factors: (i) one in which mortality was considered as the PheWAS trait, (ii) one in which borderline hypernatremia was the PheWAS trait and (iii) one in which borderline hyponatremia was the PheWAS trait. We considered ICD-10 codes associated with both mortality and dysnatremia as newly identified confounding factors. Correction for multiple tests was performed using a Bonferroni correction.

We used these new potential confounding factors to assess the association between borderline dysnatremia and in-hospital mortality. We compared different multivariate logistic models using classical confounding factors alone, confounding factors identified through the PheWAS analysis alone and both types of factors and finally chose the model with the lower Akaike Information Criterion (AIC). We retained in the final model only significantly associated variables. We provide the R code of the PheWAS analysis in Additional file 1.

All tests were two-tailed and we considered *P* values <0.05 to be significant. All statistical analyses were performed with R version 3.1.3 (RODBC, stats, MASS, MatchIt and epitools packages). This study was approved by the institutional review board of the HEGP (IRB#00001072 Study #CDW_2015_0013).

## Results

### Extraction of the patients data

**Fig. 1:**
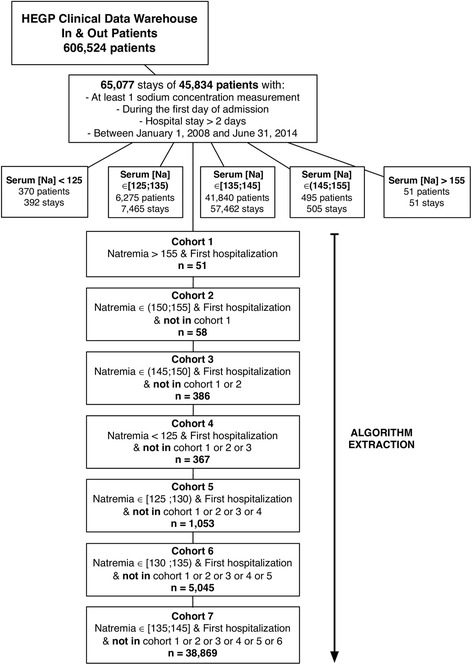
Algorithm Used to Define the Study Cohorts and Flow Chart

We extracted 606,524 patients from the CDW of the HEGP who were admitted between January 1, 2008 and June 31, 2014. At least one sodium concentration determination was available for the day of admission for 45,834 of these patients with hospital stays of longer than 2 days (Fig. 1).

### Study population

Hyponatremia and hypernatremia were recorded for 12.0% and 1.0% of hospital stays, respectively. Severe hyponatremia ([Na] < 125 mmol/L) was reported in 0.6% of hospital stays and severe hypernatremia was reported in 0.08% of hospital stays. In the vast majority of cases (57,462 hospital stays, 88.3%), serum sodium concentration was normal at admission. The mean age ± standard deviation (SD) of the patients included was 60.8 ± 18.5 years, and 59.3% of the patients were male. The mean ± SD serum sodium concentration was 138.3 ± 3.9 mmol/L. Emergency admission in hospital represented 12.1% of the cases and 20.4% of the patients spent some of their hospital stay in the ICU. Dialysis was carried out during the hospital stays for 4.4% of patients; 1.9% of the patients had been diagnosed with dementia and 1.1% were receiving palliative care. The median duration of hospital stay was 5 (3–11) days and the median number of diagnosis billing codes per stay was 4 (2–7). The median Charlson comorbidity index was 1 (0–3). The observed in-hospital mortality for the total cohort was 4.0%.

The characteristics of patients on admission to the hospital are reported in Table 1.

**Table 1:** Patient Characteristics for Each Serum Sodium Concentration at Admission Groups

Characteristic	Hyponatremia			Normal sodium level	Hypernatremia			*P* Value
Serum Sodium Level^a^, mmol/L	<125 (*n* = 367)	[125;130) (*n* = 1053)	[130;135) (*n* = 5045)	[135;145] (*n* = 38,869)	(145;150] (*n* = 386)	(150;155] (*n* = 58)	>155 (*n* = 51)	
Age, mean ± SD, years	71 ± 15	68 ± 16	66 ± 18	60 ± 18	66 ± 20	70 ± 20	73 ± 20	< .001
Males, n (%)	195 (53.1)	544 (51.6)	2916 (57.8)	23,197 (59.7)	244 (63.2)	32 (55.2)	26 (51.0)	< .001
ICU stay, n (%)	119 (32.4)	295 (28.0)	1191 (23.6)	7531 (19.4)	177 (45.9)	32 (55.2)	20 (39.2)	< .001
Palliative care, *n* (%)	11 (3.0)	45 (4.3)	151 (3.0)	296 (0.8)	14 (3.6)	1 (1.7)	4 (7.8)	< .001
Dialysis, n (%)	52 (14.2)	140 (13.3)	403 (8.0)	1322 (3.4)	48 (12.4)	6 (10.3)	6 (11.8)	< .001
Dementia, n (%)	14 (3.8)	32 (3.0)	131 (2.6)	640 (1.6)	7.3	8.6	17.6	< .001
Hospital admissions via the emergency department, *n* (%)	123 (33.5)	306 (29.1)	886 (17.6)	4091 (10.5)	95 (24.6)	22 (37.9)	25 (49.0)	< .001
Surgery, n (%)	64 (17.4)	241 (22.9)	1313 (26)	14,329 (36.9)	124 (32.1)	16 (27.6)	5 (9.8)	< .001
Number of diagnosis codes, median (IQR)	7 (4–11)	6 (4–10)	6 (3–9)	4 (2–6)	6 (4–10)	6 (3–10)	6 (4–10)	< .001
Length of stay (days), median (IQR)	10 (5–18)	10 (6–19)	8 (4–16)	5 (2–10)	9 (4–21)	10 (4.25–18.75)	10 (5–20.5)	< .001
Charlson Comorbidity Index median (IQR)	2 (1–5)	3 (1–5)	2 (1–4)	1 (0–2)	2 (0.25–3)	2 (0.25–3.75)	2 (1–3)	< .001
Observed hospital mortality, *n* (%)	69 (18.8)	145 (13.7)	452 (9.0)	1066 (2.7)	80 (20.7)	14 (24.1)	12 (23.5)	< .001

*Abbreviations*: *SD* Standard deviation, *IQR* Interquartile range, *ICU* Intensive care unit

^a^First serum sodium concentration determined the day of admission

^b^Patients admitted to the Hôpital Européen Georges Pompidou between January 1, 2008 and June 31, 2014, with at least one serum sodium concentration determined the day of admission and for hospital stays of more than two days

All the patient characteristics other than surgery with mortality were associated with both mortality and serum sodium concentration and were therefore considered to be confounding factors (Table 2).

**Table 2:** Risk of In-Hospital Mortality According to Patient and Stay Characteristics

Characteristic	OR (95%CI)	*P* Value
Serum sodium concentration, mmol/L		
< 125	7.95 (5.91–10.53)	<.001
[125;130)	5.59 (4.62–6.72)	<.001
[130;135)	3.58	<.001
[135;145]	1 [Reference]	
(145;150]	9.19 (7.06–11.83)	<.001
(150;155]	10.16 (5.10–18.74)	<.001
Age, years		
< 30	1 [Reference]	NA
[30;40)	0.66 (0.45–0.97)	.0381
[40;50)	1.02 (0.74–1.40)	.9256
[50;60)	1.94 (1.49–2.56)	<.001
[60;70)	1.83 (1.42–2.41)	<.001
[70;80)	2.43 (1.88–3.18)	<.001
[80;90)	4.00 (3.11–5.24)	<.001
≥ 90	6.41 (4.80–8.66)	<.001
Sex		
F	1 [Reference]	NA
M	1.20 (1.09–1.32)	<.001
Duration of hospital stay, days		
≤ 7	1 [Reference]	NA
(7;14]	1.59 (1.41–1.80)	<.001
(14;21]	2.55 (2.19–2.97)	<.001
(21;42]	4.04 (3.50–4.66)	<.001
> 42	5.81 (4.80–6.98)	<.001
Number of ICD-10 codes		
≤ 1	1 [Reference]	NA
(1;4]	1.39 (1.08–1.80)	.0112
(4;7]	3.96 (3.13–5.08)	<.001
(7;10]	7.34 (5.78–9.44)	<.001
(10;15]	10.61 (8.32–13.69)	<.001
(15;20]	18.31 (13.84–24.40)	<.001
> 20	31.77 (23.50–43.20)	<.001
Hospital admissions via the emergency department	2.29 (2.04–2.56)	<.001
Any surgery	1.04 (0.94–1.15)	.402
ICU stay	11.85 (10.29–12.69)	<.001
Palliative care	12.75 (10.54–15.38)	<.001
Dialysis	15.49 (13.86–17.30)	<.001
Dementia	2.67 (2.11–3.34)	<.001
Charlson Comorbidity Index		
0	1 [Reference]	NA
1	2.72 (2.21–3.36)	<.001
2	4.56 (3.76–5.54)	<.001
3–4	7.59 (6.31–9.17)	<.001
5–9	12.12 (10.11–14.62)	<.001
≥ 10	19.86 (15.31–25.72)	<.001

*Abbreviations*: *OR* Odds ratio, *CI* Confidence interval, *ICU* Intensive care unit, *ICD* International Statistical Classification of Diseases and Related Health Problems

**Fig. 2:**
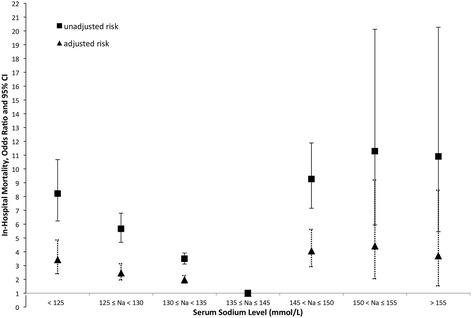
Unadjusted and Adjusted Risk of In-Hospital Mortality Estimated for Each Serum Sodium Level Group

The association between mortality and dysnatremia was highly significant, with a dose-dependent effect (Fig. 2). After adjustment for these characteristics, the association between mortality and dysnatremia remained significant (Fig. 2 and Additional file 2), with a dose-dependent effect.

The adjusted ORs for severe, moderate and borderline hyponatremia were 3.44 (95% CI, 2.41–4.86; *P* < 0.0001), 2.48 (95% CI, 1.96–3.13; *P* < 0.0001) and 1.98 (95% CI, 1.73–2.28; *P* < 0.0001), respectively. The adjusted ORs for severe, moderate and borderline hypernatremia were 4.07 (95% CI, 2.92–5.62; *P* < 0.0001), 4.42 (95% CI, 2.04–9.20; *P* < 0.0001) and 3.72 (95% CI, 1.53–8.45; *P* < 0.0001), respectively.

### Phenome-wide association analysis

Diagnoses on the studied cohort were coded with 960 different three-digits ICD-10 codes. We therefore tested the association between each ICD-10 code, mortality and natremia. This PheWAS analysis (Table 3 and Additional file 3) that identified 13 new ICD-10 codes associated with both an increased or a decreased risk of mortality and borderline hyponatremia after correction for multiple testing. Significant threshold was set at *p* < 5.10^−5^, corresponding to 0.05/960. By using the same method, the PheWAS analysis identified six new ICD-10 codes associated with both an increased or a decreased risk of mortality and borderline hypernatremia (Table 3 and Additional file 3).

**Table 3:** PheWAS Analysis: ICD-10 Codes Associate with Both Mortality and Borderline Dysnatremia

ICD-10 codes	Association with mortality OR (IC95%)	*P* Value	Association with dysnatremia OR (IC95%)	*P* Value
Boderline Hyponatremia				
A41 Other sepsis	4.7 (3.1–6.88)	< 0.001	5.83 (4.4–7.69)	< 0.001
I20 Angina pectoris	0.23 (0.13–0.37)	< 0.001	0.67 (0.55–0.79)	< 0.001
I25 Chronic ischemic heart disease	0.47 (0.34–0.64)	< 0.001	0.53 (0.44–0.62)	< 0.001
I48 Atrial fibrillation and flutter	0.15 (0.07–0.3)	< 0.001	0.62 (0.5–0.76)	< 0.001
I71 Aortic aneurysm and dissection	2.37 (1.9–2.92)	< 0.001	0.66 (0.53–0.8)	< 0.001
J15 Bacterial pneumonia, not elsewhere classified	2.9 (1.94–4.16)	< 0.001	2.75 (2.13–3.51)	< 0.001
J80 Acute respiratory distress syndrome	30.64 (23.27–40.36)	< 0.001	3.1 (2.28–4.17)	< 0.001
J96 Respiratory failure, not elsewhere classified	6.14 (5.19–7.22)	< 0.001	2.29 (1.98–2.63)	< 0.001
K65 Peritonitis	6.53 (4.46–9.3)	< 0.001	3.19 (2.32–4.33)	< 0.001
R07 Pain in throat and chest	0.1 (0.02–0.3)	< 0.001	0.35 (0.23 0.5)	< 0.001
R57 Shock, not elsewhere classified	18.73 (15.81–22.16)	< 0.001	3.56 (3.01–4.21)	< 0.001
Z48 Encounter for other postprocedural aftercare	0.63 (0.51–0.77)	< 0.001	0.62 (0.55–0.7)	< 0.001
Z51 Encounter for other aftercare	2.46 (1.97–3.04)	< 0.001	1.95 (1.68–2.25)	< 0.001
Borderline Hypernatremia				
J69 Pneumonitis due to solids and liquids	8.3 (3.53–17.2)	< 0.001	18.12 (6.74–40.49)	< 0.001
J80 Acute respiratory distress syndrome	36.98 (26.99–50.73)	< 0.001	6.96 (3.4–12.65)	< 0.001
J96 Respiratory failure, not elsewhere classified	6.88 (5.68–8.28)	< 0.001	2.95 (1.91–4.36)	< 0.001
N17 Acute kidney failure	3.33 (2.21–4.81)	< 0.001	5.84 (3.41–9.33)	< 0.001
R57 Shock, not elsewhere classified	22.39 (18.37–27.23)	< 0.001	6.19 (4.01–9.13)	< 0.001
Z51 Encounter for other aftercare	7.05 (4.32–10.98)	< 0.001	7.93 (3.7–14.89)	< 0.001

We entered these new confounders in the multivariate regression model, removed factors with P value >0.05 and chose the model that minimize the AIC. After this process, borderline hyponatremia (OR = 1.57 95% CI, 1.35–1.81; P < 0.0001) and hypernatremia (OR = 3.47 95% CI, 2.43–4.90; *P* < 0.0001) were still associated with in-hospital mortality (Table 4 and Additional file 4).

**Table 4:** Association between Dysnatremia and In-Hospital mortality. Comparison of the different regression models

Association Between Borderline Hyponatremia and Mortality			
	OR (IC95%)	P	AIC
Classical Model^a^	1.98 (1.73–2.68)	<.001	8098.4
PheWAS Model^b^	2.59 (2.28–2.94)	<.001	11,002
Final Model^c^	1.57 (1.35–1.81)	<.001	7585.5
Association Between Borderline Hypernatremia and Mortality			
	OR (IC95%)	P	AIC
Classical Model^d^	3.72(1.53–8.45)	<.001	6077.9
PheWAS Model^e^	6.23(4.60–8.33)	<.001	8761.9
Final Model^f^	3.47(2.43–4.90)	<.001	5920.8

Confounding Factors Retained in the different models:

^a^Classical model: age, duration of hospital stay, number of ICD-10 codes, hospital admissions via the emergency department, ICU stay, dialysis, palliative care, Charlson Comorbidity Index

^b^PheWAS model: A41, I20, I25, I48, I71, J15, J80, J96, K65, R07, R57, Z48, Z51

^c^Final model: classical model + I20, I25, I48, J80, R57, Z48, Z51

^d^Classical model: age, duration of hospital stay, number of ICD-10 codes, hospital admissions via the emergency department, ICU stay, dialysis, palliative care, Charlson Comorbidity Index

^e^PheWAS model: J69, J80, J96, N17, R57, S06

^f^Final model: classical model + J69, J80, R57, S06

## Discussion

Dysnatremia is one of the most frequent electrolyte disorders in medical care. It is associated with well-known morbidities and mortality. The risk of mortality is well-documented for moderate and severe dysnatremia, but recent studies have revealed a potential association between mortality and borderline dysnatremia [3–5, 18, 23, 24]. However, most of these studies concerned specific patients, from specific units and/or with specific diseases. We studied a large cohort of unselected hospitalized patients presenting the entire spectrum of dysnatremia. The data were extracted from the HEGP-CDW, a prospectively and automatically constituted database, representing real-life care data in a quaternary care hospital. After adjustment for several predefined covariables, including the Charlson comorbidity index, to take into account the complex condition of the patients, our results confirmed the findings of previous: we identified a higher risk of in-hospital mortality for patients with borderline dysnatremia. Using claims data allowed us to carry an original approach to avoid any other potential confounding factors: a PheWAS analysis. Hence, we identified new potential confounding factors without any a priori hypotheses. This approach resulted in a decrease of the observed association that confirmed the relevance of the new identified confounding factors and the association between borderline dysnatremia and in-hospital mortality. This is unprecedented, to our knowledge that such method is used for epidemiologic purpose.

The prevalence of hyponatremia at admission in patients hospitalized for at least 2 days was 12%. This estimate is close to the prevalence of 13% reported by *Waikar* et al. [3] for a comparable cohort, but lower than the 17.7% reported by Funk et al. [5] for patients admitted to the ICU. The prevalence of hypernatremia in our cohort was 1%, which is within the range of values generally reported: 0.2 to 2.5% of the general hospitalized population [34]. The patients with dysnatremia studied here were older than the general hospital population, as already reported for dysnatremia [5] and hyponatremia [3, 35]. An increase in the risk of in-hospital mortality in patients with hyponatremia has already been reported in several studies. In 2008, Zilberberg et al. [35] reported a 55% increase (OR 1.64, 95% CI 1.42–1.69) in the risk of in-hospital mortality after adjusting for various confounders in a large set of 198,281 discharge summaries. In 2009, Waikar et al. [3] reported a similar risk after multivariable adjustment, even for borderline hyponatremia (serum [Na], 130–134 mmol/L; OR 1.37, 95% CI 1.23–1.52) in a prospective cohort study of 98,411 hospitalized patients. In 2010, Wald et al. [4] confirmed these previous results in a cohort of 53,236 unselected hospitalized adults after adjustment for several confounding factors (serum [Na], 133–137 mmol/L; OR 1.34, 95% CI 1.18–1.51). These previous results and the results presented here confirm the relationship between hyponatremia and in-hospital mortality even for small variations of serum sodium concentration. The relationship between hypernatremia and in-hospital mortality has been less extensively studied. The small number of epidemiologic studies carried out may be explained by the low prevalence of hypernatremia. However, Darmon et al. [34] showed that there was a higher risk of in-hospital mortality in patients with borderline (serum [Na], 146–150 mmol/L; HR 2.03, 95% CI 1.73–2.39) to mild hypernatremia (serum [Na], > 150 mmol/L; OR 2.67, 95% CI 2.19–3.26) after adjustment for various covariables at ICU admission. This result was also confirmed by Funk et al. in a cohort of 151,486 adults consecutively admitted to an intensive care unit (serum [Na], 145–150; OR 1.48, 95% CI 1.36–1.61). Our results thus confirm, in a large set of in-hospital patients, the association of hypernatremia, even if borderline, with a higher risk of in-hospital mortality.

### Discovering new potential confounding factors using PheWAS analysis and CDW

The re-use of medical data automatically collected in the CDW allowed us a new data mining approach, a PheWAS analysis. Thus, we identified new potential confounding factors that were for all of them highly associated with both mortality and dysnatremia (Table 3). Most of them were not part of the pre-defined confounders. These new confounders cannot be taken alone in the multivariate model as they do not provide an optimal adjustment. Relationships between these new confounders and dysnatremia have to be confirmed on other datasets. However, used with the pre-defined confounders, they provided a better adjustment in the final model (Table 4). This is the first time, to our knowledge, that such an approach, combining expert knowledge and data mining approach, is used for epidemiologic purpose.

### Limitations

#### Use of procedures and billing codes as covariates

The limitations of using EHR data for epidemiologic purposes have been well described [36–38]. One of those, undercoding, may vary between diseases [39], depending on the severity of the disease and its classification as a principal or additional diagnosis. However, this bias decreases power, as patients may appear healthier than they really are. In our study, all the selected variables were associated with both dysnatremia and mortality. Another major concern is misclassification, which might have affected the attribution of patients to the different groups. The accuracy of ICD-10 billing codes has been confirmed in previous studies, particularly for the Charlson comorbidity index diagnosis codes [39, 40]. In a 2011 study based on the population-based Danish National Registry of patients, the positive predictive value of the different codes ranged from 82.0% for “diabetes mellitus with chronic complications” to 100%, for “chronic pulmonary disease” or “hemiplegia” [41]. We therefore consider this bias to be minimal and non-differential, although it was not possible to determine the extent to which this misclassification might have affected our results. The observation of an association between higher levels of the Charlson comorbidity index with higher in-hospital mortality in our cohort supports this hypothesis and consolidate that the re-use of EHR data for this kind of study is possible.

#### Low prevalence of Hypernatremia

The curve of the relationship between serum sodium concentration and mortality was U-shaped, but the U was more pronounced for hyponatremia than for hypernatremia, with a paradoxically weaker relationship for mild and severe hypernatremia than for borderline hypernatremia. This paradoxical decrease was also observed by Sakr et al.*,* in their study of 277 patients hypernatremic on admission to a surgical ICU [23]. This observation may reflect a lack of power, due to the small numbers of patients with hypernatremia in the two cohorts. This hypothesis is supported by the absence of such a paradoxical observation in the larger cohorts of Funk et al. [5] and Darmon et al. [24].

#### Causal association between Dysnatremia and in-hospital mortality

As in other studies, we identified a statistically significant association between dysnatremia and in-hospital mortality, even for small variations, but the direct contribution of dysnatremia to mortality remains unclear. The association between severe dysnatremia and morbidity can be explained by severe damage to the brain, but it remains unclear why small variations of serum sodium concentration cause morbidity and mortality. It is thus possible that dysnatremia is merely a marker of underlying disease severity, leading to death. Several studies have investigated the causal relationship between hyponatremia and mortality and have generated several hypothesis. First, hyponatremia is known to be related to higher mortality in patients with diseases activating the renin-angiotensin system and increasing vasopressin secretion, such as heart failure [8, 9], liver cirrhosis [10, 11], pulmonary embolism [12], pulmonary hypertension [13, 14], pneumonia [15, 16], and myocardial infarction [19, 20]. In this causal model, activation of the neurohumoral systems induces hyponatremia, and the associated diseases are the cause of death. However, recent studies have reconsidered this assumption: (i) Waikar et al., in their study of a prospective cohort of 98,411 patients in 2009, found that the correction of hyponatremia during hospitalization attenuated the increase in the risk of death associated with hyponatremia [3], (ii) 2 years later, the same team showed that, even for oligoanuric patients, in whom neurohumoral responses cannot influence serum sodium levels, hyponatremia was associated with an increase in the risk of mortality [17], (iii) this important result was confirmed in 2012 by Kevesdy et al., in a large cohort of 655,493 patients with chronic kidney disease in whom the relationship between dysnatremia and mortality was not dependent on the severity of kidney disease [18]. In this particular cohort, dysnatremia was related to mortality even after adjustment for multiple confounding factors.

## Conclusion

Our results confirm, by combining real-life care data and a phenome-wide association analysis, that even small variations of sodium concentration are associated with a poor prognosis. The re-use of medical data automatically collected in the CDW allowed new data mining approach. Thus, we identified new potential confounding factors that were for all of them highly associated with both mortality and dysnatremia.

## Additional files

Additional file 1: SQL code extraction from the HEGP-CDW and R Code for the PheWAS analysis (DOCX 69 kb)

Additional file 2: Unadjusted and Adjusted Risk of In-Hospital Mortality for Each Patients Subgroup According to Their Serum Sodium Concentration at Hospital Admission (DOCX 87 kb)

Additional file 3: Manhattan Plots Representing the –Log(*p*-value) of the Association Tests Between ICD-10 Billing Codes, Borderline Hyponatremia, Borderline Hypernatremia and Mortality (DOCX 85 kb)

Additional file 4: Association between Dysnatremia and In-Hospital mortality. Comparison of the different regression models (DOCX 27 kb)

## Abbreviations

AIC: Akaike Information Criterion
CDW: Clinical Data Warehouse
CKD: Chronic kidney disease
EHR: Electronic Health Records
HEGP: The Hôpital Européen Georges Pompidou
IQRs: Interquartile ranges
PheWAS: Phenome-wide association analysis
