# Supplementary material for: Association between borderline dysnatremia and mortality insight into a new data mining approach
Source: BMC Med Inform Decis Mak. 2017 Nov 22;17:152. doi: 10.1186/s12911-017-0549-7 (PMC5700671; doi:10.1186/s12911-017-0549-7)
Supplement: Supplementary file 1 — SQL code extraction from the HEGP-CDW and R Code for the PheWAS analysis (DOCX 69 kb) [file 12911_2017_549_MOESM1_ESM.docx]

**Additional File 4**

*SQL code extraction from the HEGP-CDW*

-------sélection des patients avec Natrémie > 155 à l'admission

drop table I2B2RECHDATA.YG_NA_HYPER_SEV;

create table I2B2RECHDATA.YG_NA_HYPER_SEV as

select *

from I2B2RECHDATA.YG_NA

where natremie > 155;

------sélection du premier séjour pour chaque patient

drop table I2B2RECHDATA.YG_NA_HYPER_SEV2;

create table I2B2RECHDATA.YG_NA_HYPER_SEV2 as

select y.*

from

(select patient_num, min (date_prev_na) as date_prev_na

from I2B2RECHDATA.YG_NA_HYPER_SEV

group by patient_num) A

inner join I2B2RECHDATA.YG_NA_HYPER_SEV Y

on a.date_prev_na = y.date_prev_na

and a.patient_num = y.patient_num;

-------sélection des patients avec Natrémie > 150 and <= 155 à l'admission

drop table I2B2RECHDATA.YG_NA_HYPER_MILD;

create table I2B2RECHDATA.YG_NA_HYPER_MILD as

select *

from I2B2RECHDATA.YG_NA

where natremie > 150 and patient_num not in (select patient_num from YG_NA_HYPER_SEV);

------sélection du premier séjour pour chaque patient

drop table I2B2RECHDATA.YG_NA_HYPER_MILD2;

create table I2B2RECHDATA.YG_NA_HYPER_MILD2 as

select y.*

from

(select patient_num, min (date_prev_na) as date_prev_na

from I2B2RECHDATA.YG_NA_HYPER_MILD

group by patient_num) A

inner join I2B2RECHDATA.YG_NA_HYPER_MILD Y

on a.date_prev_na = y.date_prev_na

and a.patient_num = y.patient_num;

-------sélection des patients avec Natrémie > 145 and <= 150 à l'admission

drop table I2B2RECHDATA.YG_NA_HYPER;

create table I2B2RECHDATA.YG_NA_HYPER as

select *

from I2B2RECHDATA.YG_NA

where natremie > 145 and patient_num not in (select patient_num from YG_NA_HYPER_SEV) and patient_num not in (select patient_num from YG_NA_HYPER_MILD);

------sélection du premier séjour pour chaque patient

drop table I2B2RECHDATA.YG_NA_HYPER2;

create table I2B2RECHDATA.YG_NA_HYPER2 as

select y.*

from

(select patient_num, min (date_prev_na) as date_prev_na

from I2B2RECHDATA.YG_NA_HYPER

group by patient_num) A

inner join I2B2RECHDATA.YG_NA_HYPER Y

on a.date_prev_na = y.date_prev_na

and a.patient_num = y.patient_num;

-------sélection des patients avec < 125 d'intéret lors du premier jour

drop table I2B2RECHDATA.YG_NA_HYPO_SEV;

create table I2B2RECHDATA.YG_NA_HYPO_SEV as

select *

from I2B2RECHDATA.YG_NA

where natremie < 125 and patient_num not in (select patient_num from YG_NA_HYPER_SEV) and patient_num not in (select patient_num from YG_NA_HYPER_MILD) and patient_num not in (select patient_num from YG_NA_HYPER);

------sélection du premier séjour pour chaque patient

drop table I2B2RECHDATA.YG_NA_HYPO_SEV2;

create table I2B2RECHDATA.YG_NA_HYPO_SEV2 as

select y.*

from

(select patient_num, min (date_prev_na) as date_prev_na

from I2B2RECHDATA.YG_NA_HYPO_SEV

group by patient_num) A

inner join I2B2RECHDATA.YG_NA_HYPO_SEV Y

on a.date_prev_na = y.date_prev_na

and a.patient_num = y.patient_num;

-------sélection des patients avec Natrémie >= 125 et <130 d'intéret lors du premier jour

drop table I2B2RECHDATA.YG_NA_HYPO_MILD;

create table I2B2RECHDATA.YG_NA_HYPO_MILD as

select *

from I2B2RECHDATA.YG_NA

where natremie >= 120 and natremie <130

and patient_num not in (select patient_num from YG_NA_HYPER_SEV)

and patient_num not in (select patient_num from YG_NA_HYPER_MILD)

and patient_num not in (select patient_num from YG_NA_HYPER)

and patient_num not in (select patient_num from YG_NA_HYPO_SEV);

------sélection du premier séjour pour chaque patient

drop table I2B2RECHDATA.YG_NA_HYPO_MILD2;

create table I2B2RECHDATA.YG_NA_HYPO_MILD2 as

select y.*

from

(select patient_num, min (date_prev_na) as date_prev_na

from I2B2RECHDATA.YG_NA_HYPO_MILD

group by patient_num) A

left join I2B2RECHDATA.YG_NA_HYPO_MILD Y

on a.date_prev_na = y.date_prev_na

and a.patient_num = y.patient_num;

-------sélection des patients avec Natrémie >= 130 et <135 d'intéret lors du premier jour

drop table I2B2RECHDATA.YG_NA_HYPO;

create table I2B2RECHDATA.YG_NA_HYPO as

select *

from I2B2RECHDATA.YG_NA

where natremie >= 130 and natremie <135

and patient_num not in (select patient_num from YG_NA_HYPER_SEV)

and patient_num not in (select patient_num from YG_NA_HYPER_MILD)

and patient_num not in (select patient_num from YG_NA_HYPER)

and patient_num not in (select patient_num from YG_NA_HYPO_SEV)

and patient_num not in (select patient_num from YG_NA_HYPO_MILD);

------sélection du premier séjour pour chaque patient

drop table I2B2RECHDATA.YG_NA_HYPO2;

create table I2B2RECHDATA.YG_NA_HYPO2 as

select y.*

from

(select patient_num, min (date_prev_na) as date_prev_na

from I2B2RECHDATA.YG_NA_HYPO

group by patient_num) A

left join I2B2RECHDATA.YG_NA_HYPO Y

on a.date_prev_na = y.date_prev_na

and a.patient_num = y.patient_num;

-------sélection des patients avec Natrémie >= 135 et <=145 d'intéret lors du premier jour

drop table I2B2RECHDATA.YG_NA_NORMO;

create table I2B2RECHDATA.YG_NA_NORMO as

select *

from I2B2RECHDATA.YG_NA

where natremie >= 135 and natremie <=145

and patient_num not in (select patient_num from YG_NA_HYPER_SEV)

and patient_num not in (select patient_num from YG_NA_HYPER_MILD)

and patient_num not in (select patient_num from YG_NA_HYPER)

and patient_num not in (select patient_num from YG_NA_HYPO_SEV)

and patient_num not in (select patient_num from YG_NA_HYPO_MILD)

and patient_num not in (select patient_num from YG_NA_HYPO);

------sélection du premier séjour pour chaque patient

drop table I2B2RECHDATA.YG_NA_NORMO2;

create table I2B2RECHDATA.YG_NA_NORMO2 as

select y.*

from

(select patient_num, min (date_prev_na) as date_prev_na

from I2B2RECHDATA.YG_NA_NORMO

group by patient_num) A

inner join I2B2RECHDATA.YG_NA_NORMO Y

on a.date_prev_na = y.date_prev_na

and a.patient_num = y.patient_num;

drop table I2B2RECHDATA.YG_NA2;

create table I2B2RECHDATA.YG_NA2 as

select * from YG_NA_HYPER_SEV2;

insert into I2B2RECHDATA.YG_NA2

select * from YG_NA_HYPER_MILD2;

insert into I2B2RECHDATA.YG_NA2

select * from YG_NA_HYPER2;

insert into I2B2RECHDATA.YG_NA2

select * from YG_NA_HYPO_SEV2;

insert into I2B2RECHDATA.YG_NA2

select * from YG_NA_HYPO_MILD2;

insert into I2B2RECHDATA.YG_NA2

select * from YG_NA_HYPO2;

insert into I2B2RECHDATA.YG_NA2

select * from YG_NA_NORMO2;

---------verification des duplication

SELECT encounter_num, patient_num, natremie, date_prev_na, inout_cd, start_date_sej, end_date_sej, dur_sej

FROM I2B2RECHDATA.YG_NA2

GROUP BY encounter_num, patient_num, natremie, date_prev_na, inout_cd, start_date_sej, end_date_sej, dur_sej

HAVING count(*) > 1;---------- = 0 => pas de lignes dupliquées

-----Encore plusieurs Natrémie à la même date et à la même heure

select * from I2B2RECHDATA.YG_NA2 where encounter_num in

(select encounter_num from (select distinct encounter_num, count(natremie) as natremie

from I2B2RECHDATA.YG_NA2

group by encounter_num) where natremie >= 2);

-----Selection de la valeur minimale

drop table I2B2RECHDATA.YG_NA3;

create table I2B2RECHDATA.YG_NA3 as

select y.*

from

(select patient_num, min (natremie) as natremie

from I2B2RECHDATA.YG_NA2

group by patient_num) A

inner join I2B2RECHDATA.YG_NA2 Y

on a.natremie = y.natremie

and a.patient_num = y.patient_num;

----récupération des variables âge et sex

drop table I2B2RECHDATA.YG_NA4;

create table I2B2RECHDATA.YG_NA4 as

select distinct y.*, p.death_date, floor((y.date_prev_na - p.birth_date)/365.25) as age, substr (p.sex_cd, 9, 1) as sex_cd

from I2B2DEMODATA.patient_dimension p, I2B2RECHDATA.YG_NA3 y

where p.patient_num = y.patient_num;

-----strcutures et mode d'entrée sortie

drop table I2B2RECHDATA.YG_NA_STRUCT;

create table I2B2RECHDATA.YG_NA_STRUCT as

select distinct o.encounter_num, o.patient_num, o.concept_cd as struct, o.modifier_cd as mode_ES, o.start_date, o.end_date

from i2b2demodata.observation_fact o, I2B2RECHDATA.YG_NA4 y

where o.patient_num = y.patient_num

and o.encounter_num = y.encounter_num

and o.scheme_key = 'STRUCT:';

--------ajout des décès en fonction du mode de sortie

alter table I2B2RECHDATA.YG_NA4

add DCD INT;

-------1 si décès au cour du séjour

update I2B2RECHDATA.YG_NA4

set DCD = 1

where patient_num in (select patient_num from I2B2RECHDATA.YG_NA_STRUCT where to_number (substr (mode_ES, 21, 1)) = 9);

-------0 si pas de décès au cour du séjour

update I2B2RECHDATA.YG_NA4

set DCD = 0

where patient_num not in (select patient_num from I2B2RECHDATA.YG_NA_STRUCT where to_number (substr (mode_ES, 21, 1)) = 9);

-----ajout de la variable passage en Réa

alter table I2B2RECHDATA.YG_NA_STRUCT

add REA INT;

-----REA=1 si

update I2B2RECHDATA.YG_NA_STRUCT

set REA = 1

where struct in (

'STRUCT:uh=120',

'STRUCT:uh=121',

'STRUCT:uh=36217',

'STRUCT:uh=46217',

'STRUCT:uh=46217',

'STRUCT:uh=320',

'STRUCT:uh=319',

'STRUCT:uh=321',

'STRUCT:uh=324',

'STRUCT:uh=331',

'STRUCT:uh=339',

'STRUCT:uh=340',

'STRUCT:uh=680',

'STRUCT:uh=681',

'STRUCT:uh=682',

'STRUCT:uh=683',

'STRUCT:uh=684',

'STRUCT:uh=685',

'STRUCT:uh=686');

------REA=0 si

update I2B2RECHDATA.YG_NA_STRUCT

set REA = 0

where struct not in (

'STRUCT:uh=120',

'STRUCT:uh=121',

'STRUCT:uh=36217',

'STRUCT:uh=46217',

'STRUCT:uh=46217',

'STRUCT:uh=320',

'STRUCT:uh=319',

'STRUCT:uh=321',

'STRUCT:uh=324',

'STRUCT:uh=331',

'STRUCT:uh=339',

'STRUCT:uh=340',

'STRUCT:uh=680',

'STRUCT:uh=681',

'STRUCT:uh=682',

'STRUCT:uh=683',

'STRUCT:uh=684',

'STRUCT:uh=685',

'STRUCT:uh=686');

----ajout de la variable passage en REA codée oui/non

drop table I2B2RECHDATA.YG_NA5;

create table I2B2RECHDATA.YG_NA5 as

select distinct y.*, 1 as REA

from I2B2RECHDATA.YG_NA4 y,I2B2RECHDATA.YG_NA_STRUCT s

where y.patient_num = s.patient_num

and s.rea = 1;

insert into I2B2RECHDATA.YG_NA5 (ENCOUNTER_NUM, PATIENT_NUM, NATREMIE, DATE_PREV_NA, INOUT_CD, START_DATE_SEJ, END_DATE_SEJ, DUR_SEJ, DEATH_DATE, AGE, SEX_CD, DCD, REA)

SELECT distinct ENCOUNTER_NUM, PATIENT_NUM, NATREMIE, DATE_PREV_NA, INOUT_CD, START_DATE_SEJ, END_DATE_SEJ, DUR_SEJ, DEATH_DATE, AGE, SEX_CD, DCD, 0

FROM I2B2RECHDATA.YG_NA4

where patient_num not in (select patient_num from (I2B2RECHDATA.YG_NA5));

-------création de la table avec les patients dialysés

drop table I2B2RECHDATA.YG_NA_DIAL;

create table I2B2RECHDATA.YG_NA_DIAL as

select distinct o.encounter_num, o.patient_num, o.start_date as start_dialyse, y.start_date_sej, y.end_date_sej, y.dur_sej

from i2b2demodata.observation_fact o, I2B2RECHDATA.YG_NA5 y

where o.concept_cd in (

'CIM10:Z992',

'CIM10:Z992+0',

'CIM10:Z992+1',

'CIM10:Z992+8',

'CIM10:Y841',

'CIM10:Z4921',

'CIM10:Z4920',

'CIM10:Z492',

'CIM10:Z491',

'CIM10:Z490',

'CIM10:Z49')

and o.start_date >= y.start_date_sej and o.start_date <= y.end_date_sej

and o.patient_num = y.patient_num

and o.encounter_num = y.encounter_num

and o.scheme_key = 'CIM10:';

insert into I2B2RECHDATA.YG_NA_DIAL (ENCOUNTER_NUM, PATIENT_NUM, START_DIALYSE, START_DATE_SEJ, END_DATE_SEJ, DUR_SEJ)

select distinct o.encounter_num, o.patient_num, o.start_date, y.start_date_sej, y.end_date_sej, y.dur_sej

from i2b2demodata.observation_fact o, I2B2RECHDATA.YG_NA5 y

where o.concept_cd in (

'CCAM:4657',

'CCAM:4662',

'CCAM:4656',

'CCAM:4658',

'CCAM:4653',

'CCAM:4654',

'CCAM:4660',

'CCAM:4659',

'CCAM:4661',

'CCAM:4655')

and o.start_date >= y.start_date_sej and o.start_date <= y.end_date_sej

and o.patient_num = y.patient_num

and o.encounter_num = y.encounter_num

and scheme_key = 'CCAM:';

----ajout de la variable Dialyse codée oui/non

drop table I2B2RECHDATA.YG_NA6;

create table I2B2RECHDATA.YG_NA6 as

select distinct ENCOUNTER_NUM, PATIENT_NUM, NATREMIE, DATE_PREV_NA, INOUT_CD, START_DATE_SEJ, END_DATE_SEJ, DUR_SEJ, DEATH_DATE, AGE, SEX_CD, DCD, REA, 1 as DIAL

from I2B2RECHDATA.YG_NA5

where patient_num in (select distinct patient_num from I2B2RECHDATA.YG_NA_DIAL);

insert into I2B2RECHDATA.YG_NA6 (ENCOUNTER_NUM, PATIENT_NUM, NATREMIE, DATE_PREV_NA, INOUT_CD, START_DATE_SEJ, END_DATE_SEJ, DUR_SEJ, DEATH_DATE, AGE, SEX_CD, DCD, REA, DIAL)

SELECT distinct ENCOUNTER_NUM, PATIENT_NUM, NATREMIE, DATE_PREV_NA, INOUT_CD, START_DATE_SEJ, END_DATE_SEJ, DUR_SEJ, DEATH_DATE, AGE, SEX_CD, DCD, REA, 0

FROM I2B2RECHDATA.YG_NA5

where patient_num not in (select distinct patient_num from I2B2RECHDATA.YG_NA_DIAL);

-----------Table avec Nature des DAS associés aux séjours

drop table YG_NA_DAS;

create table YG_NA_DAS as

select distinct y.patient_num, y.encounter_num, o.concept_cd, c.name_char

from i2b2demodata.observation_fact o, I2B2RECHDATA.YG_NA6 Y, i2b2demodata.concept_dimension c

where o.patient_num = y.patient_num

and o.encounter_num = y.encounter_num

and o.concept_cd = c.concept_cd

and o.start_date >= y.start_date_sej and o.start_date <= y.end_date_sej

and o.scheme_key = 'CIM10:' and o.modifier_cd != '@'

and o.tval_char = 'DAS';

---------ajout de la variable soin paliatif codé oui/non (1/0), code CIM10 : Z515

drop table I2B2RECHDATA.YG_NA7;

create table I2B2RECHDATA.YG_NA7 as

select distinct ENCOUNTER_NUM, PATIENT_NUM, NATREMIE, DATE_PREV_NA, INOUT_CD, START_DATE_SEJ, END_DATE_SEJ, DUR_SEJ, DEATH_DATE, AGE, SEX_CD, REA, DCD, DIAL, 1 as PALIA

from I2B2RECHDATA.YG_NA6

where patient_num in (select patient_num from YG_NA_DAS where concept_cd = 'CIM10:Z515');

insert into I2B2RECHDATA.YG_NA7

select distinct ENCOUNTER_NUM, PATIENT_NUM, NATREMIE, DATE_PREV_NA, INOUT_CD, START_DATE_SEJ, END_DATE_SEJ, DUR_SEJ, DEATH_DATE, AGE, SEX_CD, REA, DCD, DIAL, 0

from I2B2RECHDATA.YG_NA6

where patient_num not in (select patient_num from YG_NA_DAS where concept_cd = 'CIM10:Z515');

---------ajout de la variable démence code oui/non (1/0)

drop table I2B2RECHDATA.YG_NA8;

create table I2B2RECHDATA.YG_NA8 as

select distinct ENCOUNTER_NUM, PATIENT_NUM, NATREMIE, DATE_PREV_NA, INOUT_CD, START_DATE_SEJ, END_DATE_SEJ, DUR_SEJ, DEATH_DATE, AGE, SEX_CD, REA, DCD, DIAL, PALIA, 1 as DEMENCE

from I2B2RECHDATA.YG_NA7

where patient_num in (select patient_num from YG_NA_DAS where concept_cd in (

'CIM10:F00',

'CIM10:F000',

'CIM10:F0000',

'CIM10:F00000',

'CIM10:F00001',

'CIM10:F00002',

'CIM10:F0001',

'CIM10:F00010',

'CIM10:F00011',

'CIM10:F00012',

'CIM10:F0002',

'CIM10:F00020',

'CIM10:F00021',

'CIM10:F00022',

'CIM10:F0003',

'CIM10:F00030',

'CIM10:F00031',

'CIM10:F00032',

'CIM10:F0004',

'CIM10:F00040',

'CIM10:F00041',

'CIM10:F00042',

'CIM10:F001',

'CIM10:F0010',

'CIM10:F00100',

'CIM10:F00101',

'CIM10:F00102',

'CIM10:F0011',

'CIM10:F00110',

'CIM10:F00111',

'CIM10:F00112',

'CIM10:F0012',

'CIM10:F00120',

'CIM10:F00121',

'CIM10:F00122',

'CIM10:F0013',

'CIM10:F00130',

'CIM10:F00131',

'CIM10:F00132',

'CIM10:F0014',

'CIM10:F00140',

'CIM10:F00141',

'CIM10:F00142',

'CIM10:F002',

'CIM10:F0020',

'CIM10:F00200',

'CIM10:F00201',

'CIM10:F00202',

'CIM10:F0021',

'CIM10:F00210',

'CIM10:F00211',

'CIM10:F00212',

'CIM10:F0022',

'CIM10:F00220',

'CIM10:F00221',

'CIM10:F00222',

'CIM10:F0023',

'CIM10:F00230',

'CIM10:F00231',

'CIM10:F00232',

'CIM10:F0024',

'CIM10:F00240',

'CIM10:F00241',

'CIM10:F00242',

'CIM10:F009',

'CIM10:F0090',

'CIM10:F00900',

'CIM10:F00901',

'CIM10:F00902',

'CIM10:F0091',

'CIM10:F00910',

'CIM10:F00911',

'CIM10:F00912',

'CIM10:F0092',

'CIM10:F00920',

'CIM10:F00921',

'CIM10:F00922',

'CIM10:F0093',

'CIM10:F00930',

'CIM10:F00931',

'CIM10:F00932',

'CIM10:F0094',

'CIM10:F00940',

'CIM10:F00941',

'CIM10:F00942',

'CIM10:F01',

'CIM10:F010',

'CIM10:F0100',

'CIM10:F01000',

'CIM10:F01001',

'CIM10:F01002',

'CIM10:F0101',

'CIM10:F01010',

'CIM10:F01011',

'CIM10:F01012',

'CIM10:F0102',

'CIM10:F01020',

'CIM10:F01021',

'CIM10:F01022',

'CIM10:F0103',

'CIM10:F01030',

'CIM10:F01031',

'CIM10:F01032',

'CIM10:F0104',

'CIM10:F01040',

'CIM10:F01041',

'CIM10:F01042',

'CIM10:F011',

'CIM10:F0110',

'CIM10:F01100',

'CIM10:F01101',

'CIM10:F01102',

'CIM10:F0111',

'CIM10:F01110',

'CIM10:F01111',

'CIM10:F01112',

'CIM10:F0112',

'CIM10:F01120',

'CIM10:F01121',

'CIM10:F01122',

'CIM10:F0113',

'CIM10:F01130',

'CIM10:F01131',

'CIM10:F01132',

'CIM10:F0114',

'CIM10:F01140',

'CIM10:F01141',

'CIM10:F01142',

'CIM10:F012',

'CIM10:F0120',

'CIM10:F01200',

'CIM10:F01201',

'CIM10:F01202',

'CIM10:F0121',

'CIM10:F01210',

'CIM10:F01211',

'CIM10:F01212',

'CIM10:F0122',

'CIM10:F01220',

'CIM10:F01221',

'CIM10:F01222',

'CIM10:F0123',

'CIM10:F01230',

'CIM10:F01231',

'CIM10:F01232',

'CIM10:F0124',

'CIM10:F01240',

'CIM10:F01241',

'CIM10:F01242',

'CIM10:F013',

'CIM10:F0130',

'CIM10:F01300',

'CIM10:F01301',

'CIM10:F01302',

'CIM10:F0131',

'CIM10:F01310',

'CIM10:F01311',

'CIM10:F01312',

'CIM10:F0132',

'CIM10:F01320',

'CIM10:F01321',

'CIM10:F01322',

'CIM10:F0133',

'CIM10:F01330',

'CIM10:F01331',

'CIM10:F01332',

'CIM10:F0134',

'CIM10:F01340',

'CIM10:F01341',

'CIM10:F01342',

'CIM10:F018',

'CIM10:F0180',

'CIM10:F01800',

'CIM10:F01801',

'CIM10:F01802',

'CIM10:F0181',

'CIM10:F01810',

'CIM10:F01811',

'CIM10:F01812',

'CIM10:F0182',

'CIM10:F01820',

'CIM10:F01821',

'CIM10:F01822',

'CIM10:F0183',

'CIM10:F01830',

'CIM10:F01831',

'CIM10:F01832',

'CIM10:F0184',

'CIM10:F01840',

'CIM10:F01841',

'CIM10:F01842',

'CIM10:F019',

'CIM10:F0190',

'CIM10:F01900',

'CIM10:F01901',

'CIM10:F01902',

'CIM10:F0191',

'CIM10:F01910',

'CIM10:F01911',

'CIM10:F01912',

'CIM10:F0192',

'CIM10:F01920',

'CIM10:F01921',

'CIM10:F01922',

'CIM10:F0193',

'CIM10:F01930',

'CIM10:F01931',

'CIM10:F01932',

'CIM10:F0194',

'CIM10:F01940',

'CIM10:F01941',

'CIM10:F01942',

'CIM10:F051',

'CIM10:F067',

'CIM10:F0670',

'CIM10:F0671',

'CIM10:G30',

'CIM10:G300',

'CIM10:G301',

'CIM10:G308',

'CIM10:G309',

'CIM10:G31',

'CIM10:G310',

'CIM10:G311',

'CIM10:G312',

'CIM10:G318',

'CIM10:G319'));

insert into I2B2RECHDATA.YG_NA8

select distinct ENCOUNTER_NUM, PATIENT_NUM, NATREMIE, DATE_PREV_NA, INOUT_CD, START_DATE_SEJ, END_DATE_SEJ, DUR_SEJ, DEATH_DATE, AGE, SEX_CD, REA, DCD, DIAL, PALIA, 0

from I2B2RECHDATA.YG_NA7

where patient_num not in (select patient_num from YG_NA_DAS where concept_cd in (

'CIM10:F00',

'CIM10:F000',

'CIM10:F0000',

'CIM10:F00000',

'CIM10:F00001',

'CIM10:F00002',

'CIM10:F0001',

'CIM10:F00010',

'CIM10:F00011',

'CIM10:F00012',

'CIM10:F0002',

'CIM10:F00020',

'CIM10:F00021',

'CIM10:F00022',

'CIM10:F0003',

'CIM10:F00030',

'CIM10:F00031',

'CIM10:F00032',

'CIM10:F0004',

'CIM10:F00040',

'CIM10:F00041',

'CIM10:F00042',

'CIM10:F001',

'CIM10:F0010',

'CIM10:F00100',

'CIM10:F00101',

'CIM10:F00102',

'CIM10:F0011',

'CIM10:F00110',

'CIM10:F00111',

'CIM10:F00112',

'CIM10:F0012',

'CIM10:F00120',

'CIM10:F00121',

'CIM10:F00122',

'CIM10:F0013',

'CIM10:F00130',

'CIM10:F00131',

'CIM10:F00132',

'CIM10:F0014',

'CIM10:F00140',

'CIM10:F00141',

'CIM10:F00142',

'CIM10:F002',

'CIM10:F0020',

'CIM10:F00200',

'CIM10:F00201',

'CIM10:F00202',

'CIM10:F0021',

'CIM10:F00210',

'CIM10:F00211',

'CIM10:F00212',

'CIM10:F0022',

'CIM10:F00220',

'CIM10:F00221',

'CIM10:F00222',

'CIM10:F0023',

'CIM10:F00230',

'CIM10:F00231',

'CIM10:F00232',

'CIM10:F0024',

'CIM10:F00240',

'CIM10:F00241',

'CIM10:F00242',

'CIM10:F009',

'CIM10:F0090',

'CIM10:F00900',

'CIM10:F00901',

'CIM10:F00902',

'CIM10:F0091',

'CIM10:F00910',

'CIM10:F00911',

'CIM10:F00912',

'CIM10:F0092',

'CIM10:F00920',

'CIM10:F00921',

'CIM10:F00922',

'CIM10:F0093',

'CIM10:F00930',

'CIM10:F00931',

'CIM10:F00932',

'CIM10:F0094',

'CIM10:F00940',

'CIM10:F00941',

'CIM10:F00942',

'CIM10:F01',

'CIM10:F010',

'CIM10:F0100',

'CIM10:F01000',

'CIM10:F01001',

'CIM10:F01002',

'CIM10:F0101',

'CIM10:F01010',

'CIM10:F01011',

'CIM10:F01012',

'CIM10:F0102',

'CIM10:F01020',

'CIM10:F01021',

'CIM10:F01022',

'CIM10:F0103',

'CIM10:F01030',

'CIM10:F01031',

'CIM10:F01032',

'CIM10:F0104',

'CIM10:F01040',

'CIM10:F01041',

'CIM10:F01042',

'CIM10:F011',

'CIM10:F0110',

'CIM10:F01100',

'CIM10:F01101',

'CIM10:F01102',

'CIM10:F0111',

'CIM10:F01110',

'CIM10:F01111',

'CIM10:F01112',

'CIM10:F0112',

'CIM10:F01120',

'CIM10:F01121',

'CIM10:F01122',

'CIM10:F0113',

'CIM10:F01130',

'CIM10:F01131',

'CIM10:F01132',

'CIM10:F0114',

'CIM10:F01140',

'CIM10:F01141',

'CIM10:F01142',

'CIM10:F012',

'CIM10:F0120',

'CIM10:F01200',

'CIM10:F01201',

'CIM10:F01202',

'CIM10:F0121',

'CIM10:F01210',

'CIM10:F01211',

'CIM10:F01212',

'CIM10:F0122',

'CIM10:F01220',

'CIM10:F01221',

'CIM10:F01222',

'CIM10:F0123',

'CIM10:F01230',

'CIM10:F01231',

'CIM10:F01232',

'CIM10:F0124',

'CIM10:F01240',

'CIM10:F01241',

'CIM10:F01242',

'CIM10:F013',

'CIM10:F0130',

'CIM10:F01300',

'CIM10:F01301',

'CIM10:F01302',

'CIM10:F0131',

'CIM10:F01310',

'CIM10:F01311',

'CIM10:F01312',

'CIM10:F0132',

'CIM10:F01320',

'CIM10:F01321',

'CIM10:F01322',

'CIM10:F0133',

'CIM10:F01330',

'CIM10:F01331',

'CIM10:F01332',

'CIM10:F0134',

'CIM10:F01340',

'CIM10:F01341',

'CIM10:F01342',

'CIM10:F018',

'CIM10:F0180',

'CIM10:F01800',

'CIM10:F01801',

'CIM10:F01802',

'CIM10:F0181',

'CIM10:F01810',

'CIM10:F01811',

'CIM10:F01812',

'CIM10:F0182',

'CIM10:F01820',

'CIM10:F01821',

'CIM10:F01822',

'CIM10:F0183',

'CIM10:F01830',

'CIM10:F01831',

'CIM10:F01832',

'CIM10:F0184',

'CIM10:F01840',

'CIM10:F01841',

'CIM10:F01842',

'CIM10:F019',

'CIM10:F0190',

'CIM10:F01900',

'CIM10:F01901',

'CIM10:F01902',

'CIM10:F0191',

'CIM10:F01910',

'CIM10:F01911',

'CIM10:F01912',

'CIM10:F0192',

'CIM10:F01920',

'CIM10:F01921',

'CIM10:F01922',

'CIM10:F0193',

'CIM10:F01930',

'CIM10:F01931',

'CIM10:F01932',

'CIM10:F0194',

'CIM10:F01940',

'CIM10:F01941',

'CIM10:F01942',

'CIM10:F051',

'CIM10:F067',

'CIM10:F0670',

'CIM10:F0671',

'CIM10:G30',

'CIM10:G300',

'CIM10:G301',

'CIM10:G308',

'CIM10:G309',

'CIM10:G31',

'CIM10:G310',

'CIM10:G311',

'CIM10:G312',

'CIM10:G318',

'CIM10:G319'));

----sélection des diagnostiques reliés au séjour

drop table I2B2RECHDATA.YG_NA_NB_DAS;

create table I2B2RECHDATA.YG_NA_NB_DAS as

select y.patient_num, y.encounter_num, count (distinct o.concept_cd) as NB_DAS-------o.concept_cd, c.name_char, o.start_date, o.tval_char

from i2b2demodata.observation_fact o, I2B2RECHDATA.YG_NA8 Y----, i2b2demodata.concept_dimension c

where o.patient_num = y.patient_num

and o.encounter_num = y.encounter_num

------and o.concept_cd = c.concept_cd

and o.start_date >= y.start_date_sej and o.start_date <= y.end_date_sej

and o.scheme_key = 'CIM10:' and o.modifier_cd != '@'

and o.tval_char = 'DAS'

group by y.patient_num, y.encounter_num;

-----table avec nb de DAS et GHM

drop table I2B2RECHDATA.YG_NA9;

create table I2B2RECHDATA.YG_NA9 as

select distinct y.patient_num, y.encounter_num, y.NATREMIE, y.DATE_PREV_NA, y.INOUT_CD, y.START_DATE_SEJ, y.END_DATE_SEJ, y.DUR_SEJ, y.DEATH_DATE, y.AGE, sex_cd, y.REA, DCD, y.DIAL, y.PALIA, y.DEMENCE, o.concept_cd as GHM, d.nb_das

from I2B2RECHDATA.YG_NA8 y

left join i2b2demodata.observation_fact o

on o.patient_num = y.patient_num and o.encounter_num = y.encounter_num

left join I2B2RECHDATA.YG_NA_NB_DAS d

on d.patient_num = y.patient_num and d.encounter_num = y.encounter_num

where o.start_date >= y.start_date_sej and o.start_date <= y.end_date_sej

and o.scheme_key = 'GHM:' and o.modifier_cd = '@';

insert into I2B2RECHDATA.YG_NA9 (patient_num, encounter_num, NATREMIE, DATE_PREV_NA, INOUT_CD, START_DATE_SEJ, END_DATE_SEJ, DUR_SEJ, DEATH_DATE, AGE, SEX_CD, REA, DCD, DIAL, PALIA, DEMENCE, GHM, NB_DAS)

select distinct patient_num, encounter_num, NATREMIE, DATE_PREV_NA, INOUT_CD, START_DATE_SEJ, END_DATE_SEJ, DUR_SEJ, DEATH_DATE, AGE, sex_cd, REA, DCD, DIAL, PALIA, DEMENCE,'0', '0'

from I2B2RECHDATA.YG_NA8 where patient_num not in (select distinct patient_num from I2B2RECHDATA.YG_NA9);

-------Récupération du score de Charlson

drop table I2B2RECHDATA.YG_NA10_2;

create table I2B2RECHDATA.YG_NA10_2 as

select a.*, b.charlson

from I2B2RECHDATA.YG_NA9 A, yg_NA_CHARLSON B

where a.patient_num = b.patient_num;

-------Hospitalisation via les urgences

alter table I2B2RECHDATA.YG_NA10_2

add URG INT;

-----URG=1 si

update I2B2RECHDATA.YG_NA10_2

set URG = 1

where encounter_num in (

select encounter_num from i2b2demodata.encounter_mapping where encounter_ide in (select hsp_nda from I2B2RECHDATA.LIEN_HSP_CNS where hsp_nda in

(select encounter_ide from i2b2demodata.encounter_mapping where encounter_num in (select encounter_num from I2B2RECHDATA.YG_NA10)))

);

------URG=0 si

update I2B2RECHDATA.YG_NA10_2

set URG = 0

where encounter_num not in (

select encounter_num from i2b2demodata.encounter_mapping where encounter_ide in (select hsp_nda from I2B2RECHDATA.LIEN_HSP_CNS where hsp_nda in

(select encounter_ide from i2b2demodata.encounter_mapping where encounter_num in (select encounter_num from I2B2RECHDATA.YG_NA10)))

);

select count (distinct patient_num) from YG_NA10_2;

select * from I2B2RECHDATA.YG_NA10_2 where rownum < 1000;

select count (distinct patient_num) from I2B2RECHDATA.YG_NA where natremie < 125;

select count (distinct encounter_num) from I2B2RECHDATA.YG_NA where natremie < 125;

drop table YG_NA10_3;

create table YG_NA10_3 as

select * from YG_NA10_2

alter table I2B2RECHDATA.YG_NA10_3

add CHIR INT;

-----CHIR = 1 si GHM est en C

update I2B2RECHDATA.YG_NA10_3

set CHIR = 1

where substr (GHM, 8, 1) = 'C';

------CHIR = 0 si GHM en M ou K

update I2B2RECHDATA.YG_NA10_3

set CHIR = 0

where substr (GHM, 8, 1) in ('M','K');

select distinct CHIR, count (distinct patient_num)

from YG_NA10_3

group by CHIR

------Selection des codes diagnostiques des patients DCD avec une hyponatrémie borderline

drop table yg_temp;

create table yg_temp as

select distinct y.patient_num, y.encounter_num, o.concept_cd, c.name_char, o.tval_char, substr(y.GHM,6,2) as GRP_GHM, y.AGE-------o.concept_cd, c.name_char, o.start_date, o.tval_char

from i2b2demodata.observation_fact o, I2B2RECHDATA.YG_NA10_3 y, i2b2demodata.concept_dimension c

where o.patient_num = y.patient_num

and o.encounter_num = y.encounter_num

and o.concept_cd = c.concept_cd

and o.start_date >= y.start_date_sej and o.start_date <= y.end_date_sej

and o.scheme_key = 'CIM10:' and o.modifier_cd != '@'

and o.tval_char = 'DP'

and y.NATREMIE <135 and y.NATREMIE >=130

and y.DCD = 1

ORDER BY y.age DESC

select grp_ghm, count(distinct patient_num)

from yg_temp

group by grp_ghm;

select name_char

from i2b2demodata.concept_dimension

where concept_cd = 'GHM:G25%'

------selection des DP et natrémies pour le PheWAS

create table YG_DYSNAT_PHEWAS as

select distinct y.patient_num, y.encounter_num, y.natremie, o.concept_cd, c.name_char----, o.tval_char, substr(y.GHM,6,2) as GRP_GHM, y.AGE

from i2b2demodata.observation_fact o, I2B2RECHDATA.YG_NA10_3 y, i2b2demodata.concept_dimension c

where o.patient_num = y.patient_num

and o.encounter_num = y.encounter_num

and o.concept_cd = c.concept_cd

and o.start_date >= y.start_date_sej and o.start_date <= y.end_date_sej

and o.scheme_key = 'CIM10:' and o.modifier_cd != '@'

and o.tval_char = 'DP';

*R Code for the PheWAS analysis*

# Inclusion criteria: patients with natremia in 130-145 range

# Included variables in pop1 : patient id (PATIENT_NUM), diagnosis (CONCEPT_CD), borderline hyponatremia (bl_lower),

# borderline hypernatremia (bl_upper)

# Included variable for dd: patient id (PATIENT_NUM), vital status (DCD)

## HYPONATREMIA ANALYSIS ##

# PheWAS analysis

diag1=names(table(unique(pop1[,c(1,4)])$CONCEPT_CD))

pvalue1=matrix(data=NA,ncol=2,nrow=length(diag1))

OR_lower=matrix(data=NA,ncol=3,nrow=length(diag1))

OR_DCD=matrix(data=NA,ncol=3,nrow=length(diag1))

effectifs_lower=matrix(data=NA,ncol=4,nrow=length(diag1))

effectifs_DCD=matrix(data=NA,ncol=4,nrow=length(diag1))

for (i in 1:length(diag1)) {

pop1$tempvar=I(pop1$CONCEPT_CD==diag1[i])

pop2=pop1[order(paste(pop1$PATIENT_NUM,pop1$tempvar),decreasing=TRUE),c("PATIENT_NUM","bl_lower","tempvar")]

pop2=pop2[which(duplicated(pop2$PATIENT_NUM)==FALSE),]

pop2=merge(unique(dd[,c("PATIENT_NUM","DCD")]),pop2,by.x=1,by.y=1,all=FALSE)

pvalue1[i,1]=fisher.test(pop2$bl_lower,pop2$tempvar)$p.value

pvalue1[i,2]=fisher.test(pop2$DCD,pop2$tempvar)$p.value

effectifs_lower[i,c(1,2)]=table(pop2$bl_lower,pop2$tempvar)[1,]

effectifs_lower[i,c(3,4)]=table(pop2$bl_lower,pop2$tempvar)[2,]

effectifs_DCD[i,c(1,2)]=table(pop2$DCD,pop2$tempvar)[1,]

effectifs_DCD[i,c(3,4)]=table(pop2$DCD,pop2$tempvar)[2,]

if (table(pop2$bl_lower,pop2$tempvar)[2,2]!=0 & table(pop2$bl_lower,pop2$tempvar)[1,2]!=0) {

OR_lower[i,]=oddsratio(table(pop2$bl_lower,pop2$tempvar))$measure[2,]}

if (table(pop2$DCD,pop2$tempvar)[2,2]!=0 & table(pop2$DCD,pop2$tempvar)[1,2]!=0) {

OR_DCD[i,]=oddsratio(table(pop2$DCD,pop2$tempvar))$measure[2,]}

print("OK")

}

diag_assoc1=diag1[which(pvalue1[,1]<0.00005 & pvalue1[,2]<0.00005)]

result_lower=cbind(diag1[which(pvalue1[,1]<0.00005 & pvalue1[,2]<0.00005)],

round(pvalue1[which(pvalue1[,1]<0.00005 & pvalue1[,2]<0.00005),2],4),

round(OR_DCD[which(pvalue1[,1]<0.00005 & pvalue1[,2]<0.00005),],2),

round(pvalue1[which(pvalue1[,1]<0.00005 & pvalue1[,2]<0.00005),1],4),

round(OR_lower[which(pvalue1[,1]<0.00005 & pvalue1[,2]<0.00005),],2))

colnames(result_lower)=c("diag","pvalue_DC","OR_DC","OR_DC_inf","OR_DC_sup","pvalue_below","OR_below","OR_below_inf","OR_below_sup")

write.table(result_lower,file="OR_lower.txt",row.names=FALSE,col.names=TRUE)

# Graphe PheWAS

d=data.frame(diag1,pvalue1[,1])

names(d)=c("phenotype","p")

d$group=substr(d$phenotype,1,1)

d$groupnum=as.numeric(as.factor(d$group))

o=order(d$group,d$p,decreasing=FALSE)

d=d[o,]

d$color=rep(rainbow(20),table(d$group))

par(mfrow=c(1,3))

phenotypeManhattan(d,suggestive.line=0.05,genomewide.line=0.00005,sort.by.p=F,

sort.by.category.p=F,OR.size=F,OR.direction=F,use.color=T, size.x.labels = 15,size.y.labels = 15,

x.axis.label ="",y.axis.label="",max.y=15,title="")

# Multivariate analysis

x=do.call(rbind,tapply(as.factor(pop1$CONCEPT_CD),pop1$PATIENT_NUM,table))

x1=x[,diag_assoc1]

pop1_bis=cbind(unique(pop1[order(pop1$PATIENT_NUM),c(1,6)]),x1)

dd1=merge(dd,pop1_bis,by.x=1,by.y=1,all=FALSE)

cat(paste("+",diag_assoc1,sep=""))

# Associated diagnoses: +A41 +E26 +E66 +I10 +I20 +I25 +I48 +I71 +J15 +J80 +J96 +K65 +R07 +R57 +Z48 +Z51

# I71 and J96 were not included because they were part from Charlson score

# Multivariate models with "classical" covariates

modele1=glm(DCD~bl_lower+as.factor(DUR_SEJ_CL)+as.factor(AGE_CL)+SEX_CD+REA+DIAL+PALIA+DEMENCE+as.factor(CHARLSON_CL2)+URG+as.factor(NB_DAS_CL),data=dd1,family=binomial)

modele1=glm(DCD~bl_lower+as.factor(DUR_SEJ_CL)+as.factor(AGE_CL)+REA+DIAL+PALIA+as.factor(CHARLSON_CL2)+URG+as.factor(NB_DAS_CL),data=dd1,family=binomial)

modele1_res=t(rbind(round(exp(modele1$coefficient),2),t(round(exp(confint(modele1)),2)),

round(summary(modele1)$coefficients[,4],4)))

# Multivariate models with "classical" covariates and pheWAS covariates (final model)

modele1bis=glm(DCD~bl_lower+as.factor(DUR_SEJ_CL)+as.factor(AGE_CL)+SEX_CD+REA+DIAL+PALIA+DEMENCE+as.factor(CHARLSON_CL2)+URG+as.factor(NB_DAS_CL)

+A41 +I20 +I25 +I48 +J15 +J80 +K65 +R07 +R57 +Z48 +Z51

,data=dd1,family=binomial)

modele1bis_res=t(rbind(round(exp(modele1bis$coefficient),2),t(round(exp(confint(modele1bis)),2)),

round(summary(modele1bis)$coefficients[,4],4)))

# We only keep significant covariates

modele2=glm(DCD~bl_lower+as.factor(DUR_SEJ_CL)+as.factor(AGE_CL)+REA+DIAL+PALIA+as.factor(CHARLSON_CL2)+URG+as.factor(NB_DAS_CL)

+I20 +I25 +I48 +J80+R57 +Z48 +Z51

,data=dd1,family=binomial)

modele2_res=t(rbind(round(exp(modele2$coefficient),2),t(round(exp(confint(modele2)),2)),

round(summary(modele2)$coefficients[,4],4)))

# Multivariate models only with pheWAS covariates

modele3=glm(DCD~bl_lower+A41 +I20 +I25 +I48 +I71 +J15 +J80 +J96 +K65 +R07 +R57 +Z48 +Z51

,data=dd1,family=binomial)

modele3_res=t(rbind(round(exp(modele3$coefficient),2),t(round(exp(confint(modele3)),2)),

round(summary(modele3)$coefficients[,4],4)))

## HYPERNATREMIA ANALYSIS ##

# PheWAS analysis

pop_upper=pop[which(pop$NATREMIE>=135 & pop$NATREMIE<=150),]

diag_upper=names(table(unique(pop_upper[,c(1,4)])$CONCEPT_CD)) ##[which(table(unique(pop_upper[,c(1,4)])$CONCEPT_CD)>43*2)]

pvalue_upper=matrix(data=NA,ncol=2,nrow=length(diag_upper))

OR_upper=matrix(data=NA,ncol=3,nrow=length(diag_upper))

OR_DCD=matrix(data=NA,ncol=3,nrow=length(diag_upper))

effectifs_upper=matrix(data=NA,ncol=4,nrow=length(diag_upper))

effectifs_DCD=matrix(data=NA,ncol=4,nrow=length(diag_upper))

for (i in 1:length(diag_upper)) {

pop_upper$tempvar=I(pop_upper$CONCEPT_CD==diag_upper[i])

pop2=pop_upper[order(paste(pop_upper$PATIENT_NUM,pop_upper$tempvar),decreasing=TRUE),c("PATIENT_NUM","bl_upper","tempvar")]

pop2=pop2[which(duplicated(pop2$PATIENT_NUM)==FALSE),]

pop2=merge(unique(dd[,c("PATIENT_NUM","DCD")]),pop2,by.x=1,by.y=1,all=FALSE)

pvalue_upper[i,1]=fisher.test(pop2$bl_upper,pop2$tempvar)$p.value

pvalue_upper[i,2]=fisher.test(pop2$DCD,pop2$tempvar)$p.value

effectifs_upper[i,c(1,2)]=table(pop2$bl_upper,pop2$tempvar)[1,]

effectifs_upper[i,c(3,4)]=table(pop2$bl_upper,pop2$tempvar)[2,]

effectifs_DCD[i,c(1,2)]=table(pop2$DCD,pop2$tempvar)[1,]

effectifs_DCD[i,c(3,4)]=table(pop2$DCD,pop2$tempvar)[2,]

if (table(pop2$bl_upper,pop2$tempvar)[2,2]!=0 & table(pop2$bl_upper,pop2$tempvar)[1,2]!=0) {

OR_upper[i,]=oddsratio(table(pop2$bl_upper,pop2$tempvar))$measure[2,]}

if (table(pop2$DCD,pop2$tempvar)[2,2]!=0 & table(pop2$DCD,pop2$tempvar)[1,2]!=0) {

OR_DCD[i,]=oddsratio(table(pop2$DCD,pop2$tempvar))$measure[2,]}

print("OK")

}

diag_assoc_upper=diag_upper[which(pvalue_upper[,1]<0.00005 & pvalue_upper[,2]<0.00005)]

toto=merge(unique(pop[,c("PATIENT_NUM","CONCEPT_CD")]),unique(dd[,c("PATIENT_NUM","DCD")]),by.x=1,by.y=1,all=FALSE)

result_upper=cbind(diag_upper[which(pvalue_upper[,1]<0.00005 & pvalue_upper[,2]<0.00005)],

round(pvalue_upper[which(pvalue_upper[,1]<0.00005 & pvalue_upper[,2]<0.00005),2],4),

round(OR_DCD[which(pvalue_upper[,1]<0.00005 & pvalue_upper[,2]<0.00005),],2),

round(pvalue_upper[which(pvalue_upper[,1]<0.00005 & pvalue_upper[,2]<0.00005),1],4),

round(OR_upper[which(pvalue_upper[,1]<0.00005 & pvalue_upper[,2]<0.00005),],2))

colnames(result_upper)=c("diag","pvalue_DC","OR_DC","OR_DC_inf","OR_DC_sup","pvalue_upper","OR_upper","OR_upper_inf","OR_upper_sup")

write.table(result_upper,file="OR_upper.txt",row.names=FALSE,col.names=TRUE)

# PheWAS plot

d=data.frame(diag_upper,pvalue_upper[,1])

names(d)=c("phenotype","p")

d$group=substr(d$phenotype,1,1)

d$groupnum=as.numeric(as.factor(d$group))

o=order(d$group,d$p,decreasing=FALSE)

d=d[o,]

d$color=rep(rainbow(20),table(d$group))

phenotypeManhattan(d,suggestive.line=0.05,genomewide.line=0.00005,sort.by.p=F,sort.by.category.p=F,OR.size=F,OR.direction=F,use.color=T)

# Multivariate analysis

x=do.call(rbind,tapply(as.factor(pop_upper$CONCEPT_CD),pop_upper$PATIENT_NUM,table))

x1=x[,diag_assoc_upper]

pop1_bis=cbind(unique(pop_upper[order(pop_upper$PATIENT_NUM),c(1,7)]),x1)

dd1=merge(dd,pop1_bis,by.x=1,by.y=1,all=FALSE)

cat(paste("+",diag_assoc_upper,sep=""))

# Associated diagnosis: +J69 +J80 +J96 +N17 +R57 +S06

# J96 and N17 were not included because they were part from Charlson score

# Multivariate models with "classical" covariates

modele1u=glm(DCD~bl_upper+as.factor(DUR_SEJ_CL)+as.factor(AGE_CL)+REA+DIAL+PALIA+as.factor(CHARLSON_CL2)+URG+as.factor(NB_DAS_CL),data=dd1,family=binomial)

modele1u_res=t(rbind(round(exp(modele1u$coefficient),2),t(round(exp(confint(modele1u)),2)),

round(summary(modele1u)$coefficients[,4],4)))

# Multivariate models with "classical" and pheWAS covariates (final model)

modele1bisu=glm(DCD~bl_upper+as.factor(DUR_SEJ_CL)+as.factor(AGE_CL)+SEX_CD+REA+DIAL+PALIA+DEMENCE+as.factor(CHARLSON_CL2)+URG+as.factor(NB_DAS_CL)

+J69 +J80 +R57 +S06,data=dd1,family=binomial)

modele1bisu_res=t(rbind(round(exp(modele1bisu$coefficient),2),t(round(exp(confint(modele1bisu)),2)),

round(summary(modele1bisu)$coefficients[,4],4)))

# We only keep significant covariates

modele2u=glm(DCD~bl_upper+as.factor(DUR_SEJ_CL)+as.factor(AGE_CL)+REA+DIAL+PALIA+as.factor(CHARLSON_CL2)+URG+as.factor(NB_DAS_CL)

+J69+J80 +R57 +S06,data=dd1,family=binomial)

modele2u_res=t(rbind(round(exp(modele2u$coefficient),2),t(round(exp(confint(modele2u)),2)),

round(summary(modele2u)$coefficients[,4],4)))

# Multivariate models only with pheWAS covariates

modele3u=glm(DCD~bl_upper+J69 +J80 +J96 +N17 +R57 +S06,data=dd1,family=binomial)

modele3u_res=t(rbind(round(exp(modele3u$coefficient),2),t(round(exp(confint(modele3u)),2)),

round(summary(modele3u)$coefficients[,4],4)))

summary(glm(DCD~bl_upper+AGE+SEX_CD+A41 +I46 +J15 +J80 +J96 +K56 +K65 +N17 +R57 +S06 +Z94

,data=dd1,family=binomial))

summary(glm(DCD~bl_upper+DUR_SEJ+AGE+REA+PALIA+URG

+I46 +J96+N17+R57 +Z48

,data=dd1,family=binomial))
