# Supplementary material for: Association between borderline dysnatremia and mortality insight into a new data mining approach
Source: BMC Med Inform Decis Mak. 2017 Nov 22;17:152. doi: 10.1186/s12911-017-0549-7 (PMC5700671; doi:10.1186/s12911-017-0549-7)
Supplement: Supplementary file 2 — Unadjusted and Adjusted Risk of In-Hospital Mortality for Each Patients Subgroup According to Their Serum Sodium Concentration at Hospital Admission (DOCX 87 kb) [file 12911_2017_549_MOESM2_ESM.docx]

**Supporting Information Table 1. Unadjusted and Adjusted Risk of In-Hospital Mortality for Each Patients Subgroup According to Their Serum Sodium Concentration at Hospital Admission**

| **Serum Sodium**  **Level**^a^ | **Dysnatremia**  **Category** | **Number of**  **patients**^b^ | **OR (95%CI)** | ***P* Value** | **AOR**^c^ **(95%CI)** | ***P* Value** |
| --- | --- | --- | --- | --- | --- | --- |
| < 125 | Severe | 323 | 8.21 (6.23;10.68) | < .001 | 3.44 (2.41;4.86) | < .001 |
| 125 ≤ Na < 130 | Mild | 1,045 | 5.66 (4.69;6.80) | < .001 | 2.48 (1.96;3.13) | < .001 |
| 130 ≤ Na < 135 | Borderline | 3,875 | 3.49 (3.11;3.91) | < .001 | 1.98 (1.72;2.28) | < .001 |
| 135 ≤ Na ≤ 145 | Normal | 40,113 | 1 [Reference] | NA | 1 [Reference] | NA |
| 145 < Na ≤ 150 | Borderline | 375 | 9.27 (7.15;11.88) | < .001 | 4.07 (2.92;5.62) | < .001 |
| 150 < Na ≤ 155 | Mild | 54 | 11.28 (5.95;20.12) | < .001 | 4.43 (2.04;9.20) | .004 |
| > 155 | Severe | 49 | 10.91 (5.46;20.26) | < .001 | 3.72 (1.53;8.45) | .015 |

Abbreviations: OR, odds ratio; AOR, adjusted odds ratio

^a^ First Serum sodium concentration determined the day of admission

^b^ Patients admitted to the Hôpital Européen Georges Pompidou between January 1, 2008 and June 31, 2014, with at least one serum sodium concentration determined the day of admission and for hospital stays of more than two days.

^c^ Multivariable model including age, gender, length of stay, number of diagnosis codes, hospital admissions via the emergency department, ICU stay, paliative care, dialysis, dementia, Charlson comorbidity Index
