# Supplementary material for: Association between borderline dysnatremia and mortality insight into a new data mining approach
Source: BMC Med Inform Decis Mak. 2017 Nov 22;17:152. doi: 10.1186/s12911-017-0549-7 (PMC5700671; doi:10.1186/s12911-017-0549-7)
Supplement: Supplementary file 3 — Manhattan Plots Representing the –Log(p-value) of the Association Tests Between ICD-10 Billing Codes, Borderline Hyponatremia, Borderline Hypernatremia and Mortality (DOCX 85 kb) [file 12911_2017_549_MOESM3_ESM.docx]

**Additional File 1**

**Manhattan Plot Representing the –Log(p-value) of the Association Test Between Each ICD-10 Billing Code and Mortality.**


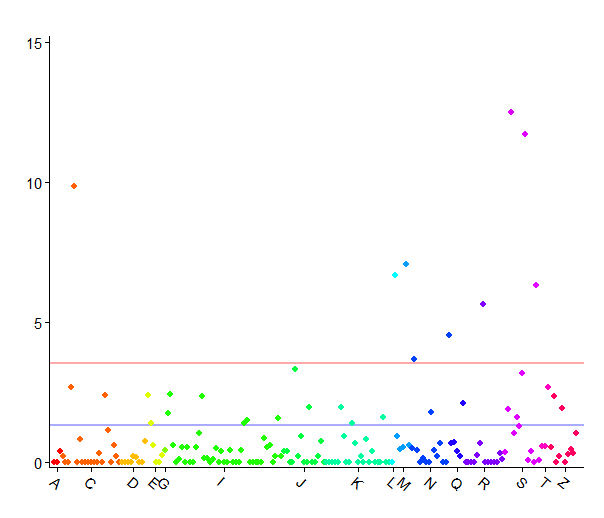


Horizontal axis represents the different ICD-10 codes ordered by their first letter and vertical axis represents the –log(p-value) for each association test. The blue horizontal line represents the 5% threshold of significance and the red line the 0.03% threshold of significance.

**Manhattan Plot Representing the –Log(p-value) of the Association Test Between Each ICD-10 Billing Code and Borderline Hyponatremia.**
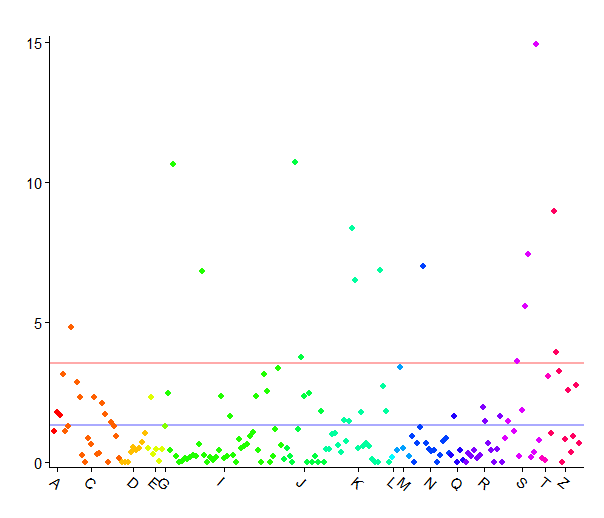


Horizontal axis represents the different ICD-10 codes ordered by their first letter and vertical axis represents the –log(p-value) for each association test. The blue horizontal line represents the 5% threshold of significance and the red line the 0.03% threshold of significance.

**Manhattan Plot Representing the –Log(p-value) of the Association Test Between each ICD-10 Billing Code and Borderline Hypernatremia.**
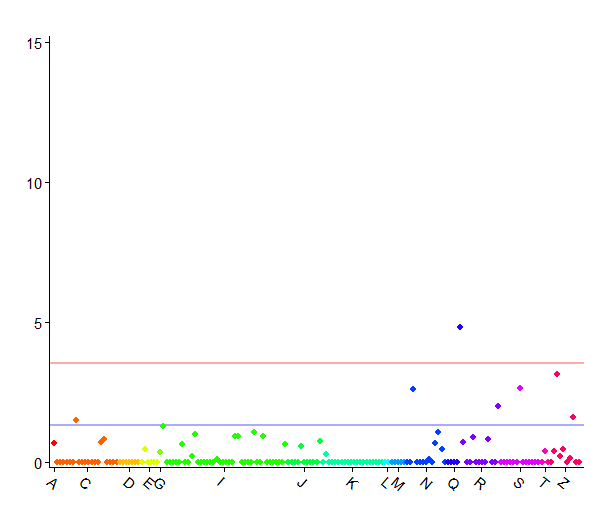


Horizontal axis represents the different ICD-10 codes ordered by their first letter and vertical axis represents the –log(p-value) for each association test. The blue horizontal line represents the 5% threshold of significance and the red line the 0.03% threshold of significance.
