# Supplementary material for: Association between borderline dysnatremia and mortality insight into a new data mining approach
Source: BMC Med Inform Decis Mak. 2017 Nov 22;17:152. doi: 10.1186/s12911-017-0549-7 (PMC5700671; doi:10.1186/s12911-017-0549-7)
Supplement: Supplementary file 4 — Association between Dysnatremia and In-Hospital mortality. Comparison of the different regression models (DOCX 27 kb) [file 12911_2017_549_MOESM4_ESM.docx]

**Association Between Borderline Hyponatremia and Mortality, Confounding Factors Retained for:**

- **Classical model:** age, duration of hospital stay, number of ICD-10 codes, hospital admissions via the emergency department, ICU stay, dialysis, palliative care, Charlson Comorbidity Index

Call:

glm(formula = DCD ~ bl_lower + as.factor(DUR_SEJ_CL) + as.factor(AGE_CL) +

REA + DIAL + PALIA + as.factor(CHARLSON_CL2) + URG + as.factor(NB_DAS_CL),

family = binomial, data = dd1)

Deviance Residuals:

Min 1Q Median 3Q Max

-2.8144 -0.1955 -0.1188 -0.0737 3.6874

Coefficients:

Estimate Std. Error z value Pr(>|z|)

(Intercept) -6.703319 0.211901 -31.634 < 2e-16 ***

bl_lowerTRUE 0.645733 0.072211 8.942 < 2e-16 ***

as.factor(DUR_SEJ_CL)2 -0.821059 0.084951 -9.665 < 2e-16 ***

as.factor(DUR_SEJ_CL)3 -1.035956 0.108181 -9.576 < 2e-16 ***

as.factor(DUR_SEJ_CL)4 -0.936474 0.107811 -8.686 < 2e-16 ***

as.factor(DUR_SEJ_CL)5 -1.086122 0.141524 -7.674 1.66e-14 ***

as.factor(AGE_CL)1 -0.177251 0.239109 -0.741 0.458512

as.factor(AGE_CL)2 0.007258 0.199624 0.036 0.970995

as.factor(AGE_CL)3 0.512261 0.172844 2.964 0.003040 **

as.factor(AGE_CL)4 0.443871 0.170632 2.601 0.009286 **

as.factor(AGE_CL)5 0.653201 0.169511 3.853 0.000116 ***

as.factor(AGE_CL)6 1.396324 0.169361 8.245 < 2e-16 ***

as.factor(AGE_CL)7 2.327369 0.200290 11.620 < 2e-16 ***

REA 2.830002 0.081543 34.706 < 2e-16 ***

DIAL 1.764359 0.081390 21.678 < 2e-16 ***

PALIA 2.862101 0.137310 20.844 < 2e-16 ***

as.factor(CHARLSON_CL2)1 0.734869 0.128644 5.712 1.11e-08 ***

as.factor(CHARLSON_CL2)2 1.157667 0.123264 9.392 < 2e-16 ***

as.factor(CHARLSON_CL2)3 1.117470 0.123324 9.061 < 2e-16 ***

as.factor(CHARLSON_CL2)4 1.647955 0.126069 13.072 < 2e-16 ***

as.factor(CHARLSON_CL2)5 2.189254 0.185263 11.817 < 2e-16 ***

URG 0.536123 0.084834 6.320 2.62e-10 ***

as.factor(NB_DAS_CL)1 0.083215 0.151014 0.551 0.581603

as.factor(NB_DAS_CL)2 0.443624 0.147672 3.004 0.002664 **

as.factor(NB_DAS_CL)3 0.687817 0.152567 4.508 6.54e-06 ***

as.factor(NB_DAS_CL)4 0.564870 0.160144 3.527 0.000420 ***

as.factor(NB_DAS_CL)5 0.688618 0.190157 3.621 0.000293 ***

as.factor(NB_DAS_CL)6 0.988231 0.209196 4.724 2.31e-06 ***

---

Signif. codes: 0 ‘***’ 0.001 ‘**’ 0.01 ‘*’ 0.05 ‘.’ 0.1 ‘ ’ 1

(Dispersion parameter for binomial family taken to be 1)

Null deviance: 12546.4 on 38317 degrees of freedom

Residual deviance: 8042.4 on 38290 degrees of freedom

(4807 observations deleted due to missingness)

AIC: 8098.4

Number of Fisher Scoring iterations: 7

- **PheWas model:** A41, I20, I25, I48, I71, J15, J80, J96, K65, R07, R57, Z48, Z51

Call:

glm(formula = DCD ~ bl_lower + A41 + I20 + I25 + I48 + I71 +

J15 + J80 + J96 + K65 + R07 + R57 + Z48 + Z51, family = binomial,

data = dd1)

Deviance Residuals:

Min 1Q Median 3Q Max

-2.9187 -0.2088 -0.2088 -0.2088 3.5940

Coefficients:

Estimate Std. Error z value Pr(>|z|)

(Intercept) -3.81488 0.03851 -99.053 < 2e-16 ***

bl_lowerTRUE 0.95355 0.06476 14.725 < 2e-16 ***

A41 0.48691 0.24362 1.999 0.0456 *

I20 -1.03767 0.26156 -3.967 7.27e-05 ***

I25 -0.33676 0.17156 -1.963 0.0496 *

I48 -1.76548 0.40583 -4.350 1.36e-05 ***

I71 1.05415 0.10215 10.320 < 2e-16 ***

J15 0.49230 0.20659 2.383 0.0172 *

J80 3.15767 0.15558 20.297 < 2e-16 ***

J96 1.32682 0.09109 14.565 < 2e-16 ***

K65 1.66876 0.18445 9.047 < 2e-16 ***

R07 -1.92825 0.70722 -2.727 0.0064 **

R57 2.62189 0.09180 28.561 < 2e-16 ***

Z48 -0.53965 0.10690 -5.048 4.46e-07 ***

Z51 0.95513 0.11465 8.331 < 2e-16 ***

---

Signif. codes: 0 ‘***’ 0.001 ‘**’ 0.01 ‘*’ 0.05 ‘.’ 0.1 ‘ ’ 1

(Dispersion parameter for binomial family taken to be 1)

Null deviance: 12943 on 43124 degrees of freedom

Residual deviance: 10972 on 43110 degrees of freedom

AIC: 11002

Number of Fisher Scoring iterations: 8

- **Final model:** classical model + I20, I25, I48, J80, R57, Z48, Z51

Call:

glm(formula = DCD ~ bl_lower + as.factor(DUR_SEJ_CL) + as.factor(AGE_CL) +

REA + DIAL + PALIA + as.factor(CHARLSON_CL2) + URG + as.factor(NB_DAS_CL) +

I20 + I25 + I48 + J80 + R57 + Z48 + Z51, family = binomial,

data = dd1)

Deviance Residuals:

Min 1Q Median 3Q Max

-2.6225 -0.1823 -0.1147 -0.0724 3.6915

Coefficients:

Estimate Std. Error z value Pr(>|z|)

(Intercept) -6.83961 0.21562 -31.720 < 2e-16 ***

bl_lowerTRUE 0.44828 0.07526 5.957 2.57e-09 ***

as.factor(DUR_SEJ_CL)2 -0.49416 0.08809 -5.609 2.03e-08 ***

as.factor(DUR_SEJ_CL)3 -0.81007 0.11244 -7.204 5.83e-13 ***

as.factor(DUR_SEJ_CL)4 -0.78300 0.11178 -7.005 2.47e-12 ***

as.factor(DUR_SEJ_CL)5 -1.04758 0.14725 -7.114 1.13e-12 ***

as.factor(AGE_CL)1 -0.09370 0.24272 -0.386 0.699467

as.factor(AGE_CL)2 0.12750 0.20384 0.625 0.531652

as.factor(AGE_CL)3 0.73166 0.17727 4.127 3.67e-05 ***

as.factor(AGE_CL)4 0.68914 0.17560 3.925 8.69e-05 ***

as.factor(AGE_CL)5 0.91834 0.17465 5.258 1.46e-07 ***

as.factor(AGE_CL)6 1.62460 0.17413 9.330 < 2e-16 ***

as.factor(AGE_CL)7 2.48182 0.20541 12.082 < 2e-16 ***

REA 3.04602 0.08698 35.020 < 2e-16 ***

DIAL 1.63772 0.08679 18.870 < 2e-16 ***

PALIA 2.88514 0.13925 20.719 < 2e-16 ***

as.factor(CHARLSON_CL2)1 0.78353 0.13212 5.931 3.02e-09 ***

as.factor(CHARLSON_CL2)2 1.00834 0.12715 7.931 2.18e-15 ***

as.factor(CHARLSON_CL2)3 0.90779 0.12773 7.107 1.18e-12 ***

as.factor(CHARLSON_CL2)4 1.26161 0.13113 9.621 < 2e-16 ***

as.factor(CHARLSON_CL2)5 1.67674 0.19382 8.651 < 2e-16 ***

URG 0.28895 0.08847 3.266 0.001090 **

as.factor(NB_DAS_CL)1 0.12081 0.15144 0.798 0.425022

as.factor(NB_DAS_CL)2 0.54279 0.14896 3.644 0.000269 ***

as.factor(NB_DAS_CL)3 0.77631 0.15509 5.006 5.57e-07 ***

as.factor(NB_DAS_CL)4 0.63771 0.16450 3.877 0.000106 ***

as.factor(NB_DAS_CL)5 0.83210 0.19833 4.196 2.72e-05 ***

as.factor(NB_DAS_CL)6 1.11337 0.22028 5.054 4.32e-07 ***

I20 -0.92555 0.28732 -3.221 0.001276 **

I25 -0.42785 0.18831 -2.272 0.023080 *

I48 -0.97602 0.42449 -2.299 0.021487 *

J80 1.34642 0.17720 7.598 3.00e-14 ***

R57 0.74817 0.10624 7.043 1.89e-12 ***

Z48 -1.50976 0.11674 -12.933 < 2e-16 ***

Z51 1.50960 0.13966 10.809 < 2e-16 ***

---

Signif. codes: 0 ‘***’ 0.001 ‘**’ 0.01 ‘*’ 0.05 ‘.’ 0.1 ‘ ’ 1

(Dispersion parameter for binomial family taken to be 1)

Null deviance: 12546.4 on 38317 degrees of freedom

Residual deviance: 7515.5 on 38283 degrees of freedom

(4807 observations deleted due to missingness)

AIC: 7585.5

Number of Fisher Scoring iterations: 8

**Association Between Borderline Hypernatremia and Mortality, Confounding factors retained for:**

- **Classical model:** age, duration of hospital stay, number of ICD-10 codes, hospital admissions via the emergency department, ICU stay, dialysis, palliative care, Charlson Comorbidity Index

Call:

glm(formula = DCD ~ bl_upper + as.factor(DUR_SEJ_CL) + as.factor(AGE_CL) +

REA + DIAL + PALIA + as.factor(CHARLSON_CL2) + URG + as.factor(NB_DAS_CL),

family = binomial, data = dd1)

Deviance Residuals:

Min 1Q Median 3Q Max

-2.9525 -0.1707 -0.0985 -0.0600 3.7706

Coefficients:

Estimate Std. Error z value Pr(>|z|)

(Intercept) -6.96155 0.23748 -29.315 < 2e-16 ***

bl_upperTRUE 1.29756 0.17534 7.400 1.36e-13 ***

as.factor(DUR_SEJ_CL)2 -1.07019 0.09917 -10.791 < 2e-16 ***

as.factor(DUR_SEJ_CL)3 -1.40098 0.13102 -10.693 < 2e-16 ***

as.factor(DUR_SEJ_CL)4 -1.23548 0.12746 -9.693 < 2e-16 ***

as.factor(DUR_SEJ_CL)5 -1.25830 0.16571 -7.593 3.12e-14 ***

as.factor(AGE_CL)1 -0.19766 0.26235 -0.753 0.451198

as.factor(AGE_CL)2 -0.15543 0.22523 -0.690 0.490136

as.factor(AGE_CL)3 0.52777 0.19022 2.775 0.005527 **

as.factor(AGE_CL)4 0.34876 0.18978 1.838 0.066107 .

as.factor(AGE_CL)5 0.71768 0.18781 3.821 0.000133 ***

as.factor(AGE_CL)6 1.47705 0.18763 7.872 3.49e-15 ***

as.factor(AGE_CL)7 2.51917 0.22823 11.038 < 2e-16 ***

REA 3.20457 0.09951 32.205 < 2e-16 ***

DIAL 1.89723 0.09531 19.907 < 2e-16 ***

PALIA 3.17978 0.17131 18.562 < 2e-16 ***

as.factor(CHARLSON_CL2)1 0.74537 0.14556 5.121 3.04e-07 ***

as.factor(CHARLSON_CL2)2 1.21202 0.13885 8.729 < 2e-16 ***

as.factor(CHARLSON_CL2)3 1.26665 0.13975 9.064 < 2e-16 ***

as.factor(CHARLSON_CL2)4 1.67316 0.14681 11.397 < 2e-16 ***

as.factor(CHARLSON_CL2)5 2.20956 0.23011 9.602 < 2e-16 ***

URG 0.67667 0.09885 6.846 7.61e-12 ***

as.factor(NB_DAS_CL)1 0.05117 0.17722 0.289 0.772774

as.factor(NB_DAS_CL)2 0.44160 0.17379 2.541 0.011055 *

as.factor(NB_DAS_CL)3 0.72080 0.17988 4.007 6.15e-05 ***

as.factor(NB_DAS_CL)4 0.66249 0.18852 3.514 0.000441 ***

as.factor(NB_DAS_CL)5 0.59730 0.23009 2.596 0.009433 **

as.factor(NB_DAS_CL)6 0.87074 0.25095 3.470 0.000521 ***

---

Signif. codes: 0 ‘***’ 0.001 ‘**’ 0.01 ‘*’ 0.05 ‘.’ 0.1 ‘ ’ 1

(Dispersion parameter for binomial family taken to be 1)

Null deviance: 9784.5 on 33962 degrees of freedom

Residual deviance: 6021.9 on 33935 degrees of freedom

(4627 observations deleted due to missingness)

AIC: 6077.9

Number of Fisher Scoring iterations: 8

- **PheWas model:** J69, J80, J96, N17, R57, S06

Call:

glm(formula = DCD ~ bl_upper + J69 + J80 + J96 + N17 + R57 +

S06, family = binomial, data = dd1)

Deviance Residuals:

Min 1Q Median 3Q Max

-2.8275 -0.1984 -0.1984 -0.1984 2.8065

Coefficients:

Estimate Std. Error z value Pr(>|z|)

(Intercept) -3.91861 0.03729 -105.090 < 2e-16 ***

bl_upperTRUE 1.82972 0.15139 12.086 < 2e-16 ***

J69 1.41651 0.48482 2.922 0.00348 **

J80 3.37609 0.17570 19.215 < 2e-16 ***

J96 1.56784 0.10254 15.290 < 2e-16 ***

N17 1.11375 0.18885 5.898 3.69e-09 ***

R57 2.95341 0.10589 27.892 < 2e-16 ***

S06 1.59494 0.20256 7.874 3.44e-15 ***

---

Signif. codes: 0 ‘***’ 0.001 ‘**’ 0.01 ‘*’ 0.05 ‘.’ 0.1 ‘ ’ 1

(Dispersion parameter for binomial family taken to be 1)

Null deviance: 10122.0 on 38589 degrees of freedom

Residual deviance: 8745.9 on 38582 degrees of freedom

AIC: 8761.9

Number of Fisher Scoring iterations: 6

- **Final model:** classical model + J69, J80, R57, S06

Call:

glm(formula = DCD ~ bl_upper + as.factor(DUR_SEJ_CL) + as.factor(AGE_CL) +

REA + DIAL + PALIA + as.factor(CHARLSON_CL2) + URG + as.factor(NB_DAS_CL) +

J69 + J80 + R57 + S06, family = binomial, data = dd1)

Deviance Residuals:

Min 1Q Median 3Q Max

-2.8105 -0.1691 -0.0991 -0.0614 3.7556

Coefficients:

Estimate Std. Error z value Pr(>|z|)

(Intercept) -6.97306 0.24205 -28.809 < 2e-16 ***

bl_upperTRUE 1.23154 0.17921 6.872 6.32e-12 ***

as.factor(DUR_SEJ_CL)2 -0.99525 0.10064 -9.890 < 2e-16 ***

as.factor(DUR_SEJ_CL)3 -1.39623 0.13440 -10.388 < 2e-16 ***

as.factor(DUR_SEJ_CL)4 -1.20216 0.13056 -9.207 < 2e-16 ***

as.factor(DUR_SEJ_CL)5 -1.29082 0.17112 -7.543 4.58e-14 ***

as.factor(AGE_CL)1 -0.12558 0.26621 -0.472 0.637106

as.factor(AGE_CL)2 -0.13424 0.22982 -0.584 0.559141

as.factor(AGE_CL)3 0.55397 0.19419 2.853 0.004334 **

as.factor(AGE_CL)4 0.39930 0.19376 2.061 0.039326 *

as.factor(AGE_CL)5 0.76817 0.19183 4.004 6.22e-05 ***

as.factor(AGE_CL)6 1.53897 0.19127 8.046 8.56e-16 ***

as.factor(AGE_CL)7 2.56910 0.23165 11.090 < 2e-16 ***

REA 3.01981 0.10167 29.702 < 2e-16 ***

DIAL 1.87490 0.09815 19.101 < 2e-16 ***

PALIA 3.23150 0.17107 18.890 < 2e-16 ***

as.factor(CHARLSON_CL2)1 0.78662 0.14769 5.326 1.00e-07 ***

as.factor(CHARLSON_CL2)2 1.21332 0.14118 8.594 < 2e-16 ***

as.factor(CHARLSON_CL2)3 1.27361 0.14235 8.947 < 2e-16 ***

as.factor(CHARLSON_CL2)4 1.61417 0.15047 10.728 < 2e-16 ***

as.factor(CHARLSON_CL2)5 2.12984 0.23975 8.883 < 2e-16 ***

URG 0.61351 0.10093 6.079 1.21e-09 ***

as.factor(NB_DAS_CL)1 0.04713 0.17744 0.266 0.790537

as.factor(NB_DAS_CL)2 0.40652 0.17446 2.330 0.019800 *

as.factor(NB_DAS_CL)3 0.63868 0.18137 3.521 0.000429 ***

as.factor(NB_DAS_CL)4 0.53608 0.19081 2.809 0.004962 **

as.factor(NB_DAS_CL)5 0.39881 0.23534 1.695 0.090142 .

as.factor(NB_DAS_CL)6 0.55225 0.25888 2.133 0.032904 *

J69 0.72576 0.53487 1.357 0.174817

J80 1.81563 0.20555 8.833 < 2e-16 ***

R57 1.10541 0.12582 8.786 < 2e-16 ***

S06 1.30311 0.21879 5.956 2.58e-09 ***

---

Signif. codes: 0 ‘***’ 0.001 ‘**’ 0.01 ‘*’ 0.05 ‘.’ 0.1 ‘ ’ 1

(Dispersion parameter for binomial family taken to be 1)

Null deviance: 9784.5 on 33962 degrees of freedom

Residual deviance: 5856.8 on 33931 degrees of freedom

(4627 observations deleted due to missingness)

AIC: 5920.8

Number of Fisher Scoring iterations: 8
